# Incidence of Total Knee Arthroplasty After Arthroscopic Surgery for Knee Osteoarthritis: A Secondary Analysis of a Randomized Clinical Trial

**DOI:** 10.1001/jamanetworkopen.2024.6578

**Published:** 2024-04-18

**Authors:** Trevor B. Birmingham, Codie A. Primeau, Salimah Z. Shariff, Jennifer N. S. Reid, Jacquelyn D. Marsh, Melody Lam, Stephanie N. Dixon, J. Robert Giffin, Kevin R. Willits, Robert B. Litchfield, Brian G. Feagan, Peter J. Fowler

**Affiliations:** 1 Fowler Kennedy Sport Medicine Clinic, University of Western Ontario, London, Canada; 2 School of Physical Therapy, Faculty of Health Sciences, University of Western Ontario, London, Canada; 3 Bone and Joint Institute, University of Western Ontario, London, Canada; 4 ICES Western, Lawson Health Research Institute, London Health Sciences Centre, London, Ontario, Canada; 5 Arthur Labatt Family School of Nursing, Faculty of Health Sciences, University of Western Ontario, London, Canada; 6 Department of Epidemiology and Biostatistics, Schulich School of Medicine and Dentistry, University of Western Ontario, London, Canada; 7 Department of Surgery, Schulich School of Medicine and Dentistry, University of Western Ontario, London, Canada; 8 Department of Medicine, Schulich School of Medicine and Dentistry, University of Western Ontario, London, Canada

## Abstract

**Importance:**

It is unclear whether arthroscopic resection of degenerative knee tissues among patients with osteoarthritis (OA) of the knee delays or hastens total knee arthroplasty (TKA); opposite findings have been reported.

**Objective:**

To compare the long-term incidence of TKA in patients with OA of the knee after nonoperative management with or without additional arthroscopic surgery.

**Design, Setting, and Participants:**

In this ad hoc secondary analysis of a single-center, assessor-blinded randomized clinical trial performed from January 1, 1999, to August 31, 2007, 178 patients were followed up through March 31, 2019. Participants included adults diagnosed with OA of the knee referred for potential arthroscopic surgery in a tertiary care center specializing in orthopedics in London, Ontario, Canada. All participants from the original randomized clinical trial were included. Data were analyzed from June 1, 2021, to October 20, 2022.

**Exposures:**

Arthroscopic surgery (resection or debridement of degenerative tears of the menisci, fragments of articular cartilage, or chondral flaps and osteophytes that prevented full extension) plus nonoperative management (physical therapy plus medications as required) compared with nonoperative management only (control).

**Main Outcomes and Measures:**

Total knee arthroplasty was identified by linking the randomized trial data with prospectively collected Canadian health administrative datasets where participants were followed up for a maximum of 20 years. Multivariable Cox proportional hazards regression models were used to compare the incidence of TKA between intervention groups.

**Results:**

A total of 178 of 277 eligible patients (64.3%; 112 [62.9%] female; mean [SD] age, 59.0 [10.0] years) were included. The mean (SD) body mass index was 31.0 (6.5). With a median follow-up of 13.8 (IQR, 8.4-16.8) years, 31 of 92 patients (33.7%) in the arthroscopic surgery group vs 36 of 86 (41.9%) in the control group underwent TKA (adjusted hazard ratio [HR], 0.85 [95% CI, 0.52-1.40]). Results were similar when accounting for crossovers to arthroscopic surgery (13 of 86 [15.1%]) during follow-up (HR, 0.88 [95% CI, 0.53-1.44]). Within 5 years, the cumulative incidence was 10.2% vs 9.3% in the arthroscopic surgery group and control group, respectively (time-stratified HR for 0-5 years, 1.06 [95% CI, 0.41-2.75]); within 10 years, the cumulative incidence was 23.3% vs 21.4%, respectively (time-stratified HR for 5-10 years, 1.06 [95% CI, 0.45-2.51]). Sensitivity analyses yielded consistent results.

**Conclusions and Relevance:**

In this secondary analysis of a randomized clinical trial of arthroscopic surgery for patients with OA of the knee, a statistically significant association with delaying or hastening TKA was not identified. Approximately 80% of patients did not undergo TKA within 10 years of nonoperative management with or without additional knee arthroscopic surgery.

**Trial Registration:**

ClinicalTrials.gov Identifier: NCT00158431

## Key Points

**Question:**

Among patients with knee osteoarthritis (OA), does the addition of arthroscopic surgery (resection of degenerative knee tissues, including meniscal tears) to nonoperative management affect the long-term incidence of total knee arthroplasty (TKA)?

**Findings:**

In this secondary analysis of a randomized clinical trial of 178 participants, there was no significant difference in the cumulative incidence of TKA between the arthroscopic surgery and control groups.

**Meaning:**

These findings suggest that the addition of arthroscopic surgery to nonoperative management for patients with OA of the knee did not delay or hasten TKA.

## Teaser

This extended follow-up of a randomized clinical trial compares the long-term incidence of total knee arthroplasty on the study knee between patients undergoing arthroscopic surgery and nonsurgical management for osteoarthritis.

## Introduction

Knee arthroscopic surgery and total knee arthroplasty (TKA) among patients with knee osteoarthritis (OA) are 2 of the most common orthopedic procedures internationally, with patients frequently undergoing both operations.^1,2,3,4,5,6^ Arthroscopic surgery for patients with OA of the knee includes removing the degenerative portions of the menisci, articular chondral fragments, and osteophytes that are thought to contribute to symptoms during a single operation.^7,8,9,10^ As partial meniscectomy is known to increase the risk of the development and progression of joint damage,^11,12,13,14,15,16^ the aim of arthroscopic surgery for patients with OA of the knee is to resect as little tissue as possible while removing the degenerative portions that contribute to symptoms.^7,8,9,10^

Case series suggest arthroscopic surgery can delay more invasive operations for OA of the knee, such as TKA or osteotomy (limb realignment), and this message is repeated in peer-reviewed journal articles and patient information materials.^17,18,19,20,21,22^ Conversely, data from longitudinal cohort studies suggest arthroscopic surgery among patients with or at risk of developing OA of the knee may increase the incidence of TKA when compared with similar patients who did not receive arthroscopic surgery.^23,24^ Clinical practice guidelines for OA do not address this discrepancy.^25,26,27,28^

There are several systematic reviews and recommendations regarding arthroscopic surgery for degenerative knee disease.^29,30,31,32,33^ Rates of arthroscopic surgery among patients with OA of the knee remain high internationally,^6,29,34,35,36,37,38,39,40^ including during the years directly preceding TKA.^5,6,41^ Rates of TKA are increasing markedly, vary considerably by region, and may be influenced by several factors related to the patients, clinicians, and health systems.^2,42,43,44,45,46,47,48,49,50^ Controlling for these factors in studies is challenging, especially in observational designs,^51,52^ and may result in overestimating a causal effect of knee arthroscopic surgery on delaying or hastening TKA.

In a randomized clinical trial conducted by members of our group, Kirkley et al^8^ reported results for patients with OA of the knee assigned to arthroscopic surgery plus nonoperative management (arthroscopic surgery group) compared with nonoperative management only (control group). The results indicated no additional benefit of surgery on pain, stiffness, or function at 2 years of follow-up.^8^ The study was completed at a single orthopedic center providing surgical and nonoperative treatments for OA of the knee within a universal health system where procedures are prospectively recorded in government health administrative datasets. This provided the opportunity to investigate the effect of arthroscopic surgery for OA of the knee on the long-term incidence of TKA using a randomized clinical trial design. In the present analysis, our primary objective was to compare the long-term incidence of TKA on the study knee between the arthroscopic surgery and control groups. Our secondary objective was to compare the long-term incidence of TKA or high tibial osteotomy on either knee.

## Methods

### Study Design

The single-center, assessor-blinded, parallel-group randomized clinical trial was performed between January 1, 1999, and August 31, 2007, at the Fowler Kennedy Sport Medicine Clinic, London, Ontario, Canada, with results published in 2008^8^ and 2016.^53^ The trial data were linked with Canadian provincial health care datasets held at ICES (formerly the Institute for Clinical Evaluation Sciences)^54^ to determine the long-term incidence of TKA or high tibial osteotomy in trial participants by extending the maximum follow-up date to March 31, 2019. In this secondary analysis, we analyzed prospectively collected ICES data in accordance with a dataset creation and statistical analysis plan completed prior to the analysis; however, this analysis was not specified in our original trial protocol (found in Supplement 1). ICES is an independent, not-for-profit research institute funded by an annual grant from the Ontario Ministry of Health and the Ministry of Long-Term Care. As a prescribed entity under Ontario’s privacy legislation, ICES is authorized to collect and use health care data for the purposes of health system analysis, evaluation, and decision support. Secure access to these data is governed by policies and procedures permitted by the Information and Privacy Commissioner of Ontario. The original trial and extended follow-up were approved by the Research Ethics Board at the University of Western Ontario, London, including a waiver of consent to access administrative health data for research. We followed the Consolidated Standards of Reporting Trials statement extension for trials conducted using cohorts and routinely collected data (CONSORT-ROUTINE) reporting guideline for randomized clinical trials conducted using administrative data.

### Data Sources

Data previously collected for the trial^8,53^ included demographic and clinical characteristics, randomization, and treatment details, as well as patient-reported outcome measures. The trial data were supplemented with the following health administrative datasets at ICES: the Canadian Institute for Health Information’s Discharge Abstract Database and Same-Day Surgery Database, which capture hospitalizations and same-day surgical procedures, respectively; the Ontario Health Insurance Plan database, which captures outpatient visits; and the Registered Persons Database, which contains demographic information (eTable 1 in Supplement 2). The accuracy and completeness of data from these sources have been confirmed for hospital-based interventions, such as the incidence of TKA.^55,56^ The datasets were linked using unique encoded identifiers and analyzed at ICES.

### Participants

All participants from the original trial were included in our study. Patients were 18 years or older, had a diagnosis of OA of the knee,^57^ and had a radiograph Kellgren and Lawrence (KL) grade of 2 or greater (KL grades range from 0 to 4, where higher grades represent greater severity).^58^ Racial and ethnic data were not collected as part of the original study. Exclusion criteria consisted of inflammatory or postinfectious arthritis, previous arthroscopic surgery of the knee, more than 5 degrees of varus or valgus malalignment, previous major knee trauma, late-stage radiographic disease (KL grade 4) with 2 or more knee compartments affected in persons older than 60 years, intra-articular corticosteroid injection within the previous 3 months, a major neurological deficit, serious medical illness (life expectancy of <2 years or high intraoperative risk), and pregnancy. Patients with a traumatic, mechanical tear of the meniscus (eg, bucket handle) detected by history and results of physical examination^59,60,61^ were not eligible. Patients who were unable to provide informed consent or who were deemed unlikely to comply with follow-up were excluded.

### Interventions

Patients were randomized to the arthroscopic surgery or the control group using varying block sizes of 2 and 4. Randomization was also stratified by surgeon and radiographic disease stage (KL grade 2 vs ≥3).^58^ Knee arthroscopy was performed using general anesthesia and a tourniquet and thigh holder. The orthopedic surgeon evaluated the medial, lateral, and patellofemoral compartments, irrigated the knee with at least 1 L of saline, and performed 1 or more of the following: resection or debridement of degenerative tears of the menisci, fragments of articular cartilage, and/or chondral flaps and osteophytes that prevented full extension and/or synovectomy.^8^ Within 7 days after surgery, the patient began the same nonoperative treatments as patients assigned to the control group.

Nonoperative management included physical and medical therapy according to evidence-based guidelines that emphasized education and exercise, which remain the core nonoperative treatments for OA of the knee.^62,63^ Patients were provided with weekly 1-hour individual sessions with a physiotherapist over 12 consecutive weeks. After patients had completed 12 weeks of supervised exercises, they were instructed to continue the program at home. The patients reviewed their medical treatment plans with an orthopedic surgeon, and medications were optimized according to stepwise use of acetaminophen and nonsteroidal anti-inflammatory drugs.

### Outcome Measures

The primary outcome was TKA on the study knee, defined using Canadian Classification of Health Interventions codes found in the Discharge Abstract Database and Same-Day Surgery Database (eTable 2 in Supplement 2). Laterality of TKA was missing for 5 or fewer patients and was assumed to be on the study knee. The secondary outcome was TKA or osteotomy on either knee. Osteotomy was defined using Ontario Health Insurance Plan database billing records, which do not indicate laterality. Osteotomy was performed on 5 or fewer patients. In accordance with ICES privacy policies, details for cell sizes less than or equal to 5 have been suppressed.

### Statistical Analysis

Statistical analysis was completed from June 1, 2021, to October 20, 2022. In the primary intention-to-treat analysis, the crude event rate (percentage) of TKA and event rate per 100 person-years were evaluated. We generated cumulative incidence curves (complement of Kaplan-Meier) as a function of time by treatment group. Patients were followed up from the date of randomization until March 31, 2019, for study outcomes and were censored on emigration from the province (ie, no health care contact within 5 years) or death. We fit a multivariable cause-specific Cox proportional hazards regression model to evaluate the association between treatment group and TKA, adjusting for the following covariates defined a priori: baseline age (per 10 years), sex, body mass index (calculated as weight in kilograms divided by height in meters squared) per 5 U, KL grade 2 vs 3 or greater, and total score on the Western Ontario and McMaster Universities OA Index (WOMAC) (per 200 points). The WOMAC is scored from 0 to 2400, with higher values indicating greater pain and stiffness and reduced function.^64^ We assessed the proportional hazards assumption using Schoenfeld residuals and an interaction with time.

We conducted additional analyses to evaluate the robustness of our findings. We repeated the primary Cox proportional hazards regression model while including knee arthroscopic surgery received during the long-term follow-up (ie, crossovers from control to arthroscopic surgery) as a time-varying covariate. Consistent with the previous analyses for this trial, we further repeated the primary analysis while including the 6 patients who were randomized to but did not receive arthroscopic surgery in the control group (as-treated analysis) and performed 2 subgroup analyses. For patients with KL grade 2, we evaluated the association between treatment group and TKA, adjusting for age. For patients reporting catching or locking of the knee, we evaluated the association between treatment group and TKA adjusting for age, sex, and radiographic severity. These analyses were repeated for the primary and secondary outcome measures. Last, we completed 2 post hoc analyses on the primary and secondary outcomes after our data analysis plan was developed to enable better comparison of our results to previously reported results at approximately 5 and 10 years.^23,29,65^ We completed a time-stratified Cox proportional hazards regression model (ie, an extended Cox model using Heaviside functions) to generate adjusted hazard ratios (HRs) within less than 5, 5 to 10, and more than 10 years since randomization. Analyses were performed using SAS, version 9.4 (SAS Institute Inc). Two-sided tests with a type I error rate of .05 were used for the primary analysis (*P* < .05 indicating statistical significance). All subsequent analyses report point estimates and corresponding 95% CIs (unadjusted for multiple testing) and should be considered exploratory.^66^

## Results

A total of 178 of 277 eligible patients (64.3%; 112 [62.9%] female and 66 [37.1%] male; mean [SD] age, 59.0 [10.0] years; mean [SD] body mass index, 31.0 [6.5]) were included in this study; 89 were ineligible or declined participation and 10 withdrew consent after randomization. Between January 1, 1999, and August 31, 2005 (with initial trial follow-up to August 31, 2007), 178 patients were randomly assigned to the arthroscopic surgery (n = 92) or the control (n = 86) groups. The eFigure in Supplement 2 shows the patient flow diagram. Baseline characteristics are summarized in Table 1 and with greater detail in eTable 3 in Supplement 2. Patients were primarily middle-aged and had overweight and KL grades of 2 or 3. Arthroscopic procedures and nonoperative treatments received over the initial 2-year follow-up have been previously reported and are repeated in eTable 4 in Supplement 2. Of the 92 patients randomized to arthroscopic surgery, 6 declined the procedure. Partial resection or debridement of degenerative tears of the meniscus was performed in 70 of 86 patients (81.4%). Debridement of degenerative articular cartilage fragments was performed in 83 of 86 patients (96.5%).

**Table 1.** Baseline Demographic and Clinical Characteristics^a^

Characteristic	Patient group			
	Arthroscopic surgery (n = 92)	Control (n = 86)	Total (N = 178)
Age, y			
Mean (SD)	58.1 (10.1)	59.9 (9.9)	59.0 (10.0)
Median (IQR)	57 (52-66)	61 (54-67)	59 (52-66)
Sex, No. (%)			
Female	54 (58.7)	58 (67.4)	112 (62.9)
Male	38 (41.3)	28 (32.6)	66 (37.1)
Body mass index^b^			
Mean (SD)	31.6 (6.7)	30.2 (6.3)	31.0 (6.5)
Median (IQR)	31 (27-35)	29 (25-34)	30 (26-35)
Kellgren and Lawrence grade, No. (%)^c^			
2	≤45	36 (41.9)	≤80
3	45 (48.9)	45 (52.3)	90 (50.6)
4	≤5	≤5	9 (5.1)
Missing	≤5	≤5	≤5
Baseline WOMAC total score^d^			
Mean (SD)	1169.95 (482.58)	1066.46 (551.39)	1120.56 (517.67)
Median (IQR)	1184 (811-1497)	1102 (665-1546)	1154 (759-1503)

Abbreviation: WOMAC, Western Ontario and McMaster Universities Osteoarthritis Index.

^a^ All patients had a clinical diagnosis of osteoarthritis of the knee according to the American College of Rheumatology Criteria as described by Altman et al.^57^ Clinical osteoarthritis of the knee is defined as knee pain and 3 of the 6 following criteria: morning stiffness less than 30 minutes, older than 50 years, crepitus, bony tenderness, bony enlargement, and/or no palpable warmth.

^b^ Calculated as weight in kilograms divided by height in meters squared.

^c^ Higher grades indicate greater severity on radiograph.^58^ In accordance with ICES privacy policies, cell sizes less than or equal to 5 cannot be reported.

^d^ Includes 3 subscales (pain, stiffness, and physical function) calculated from 24 questions. Values range from 0 (no disease) to 2400 (more severe disease).

Participants were followed up for as long as 20 years, with a median of 13.8 (IQR, 8.4-16.8) years. The follow-up periods were similar across both groups: median of 13.9 (IQR, 8.2-16.9) years in the arthroscopic surgery group and 13.6 (IQR, 8.4-16.8) years for the control group. Fourteen participants in each of the treatment groups died prior to the end of follow-up. At the end of follow-up, 67 of the 178 patients (37.6%) underwent TKA, including 31 of the 92 patients (33.7%; 2.71 per 100 person-years) in the arthroscopic surgery group and 36 of the 86 patients (41.9%; 3.38 per 100 person-years) in the control group (Table 2).

**Table 2.** Descriptive Statistics for Primary and Secondary Outcomes

Outcome	TKA on study knee by study group	
	Arthroscopic surgery (n = 92)	Control (n = 86)
**TKA on study knee (primary outcome)**		
Events, No. (%)	31 (33.7)	36 (41.9)
Event rate per 100 person-years	2.71	3.38
Deaths, No. (%)	14 (15.2)	14 (16.3)
Loss to follow-up, No. (%)	≤5^a^	0
**TKA or high tibial osteotomy on either knee (secondary outcome)**		
Events, No. (%)	41 (44.6)	46 (53.5)
Event rate per 100 person-years	4.00	4.62
Deaths, No. (%)	14 (15.2)	12 (14.0)
Loss to follow-up, No. (%)	≤5^a^	0

Abbreviation: TKA, total knee arthroplasty.

^a^ In accordance with ICES privacy policies, cell sizes less than or equal to 5 cannot be reported.

Cumulative incidence (complement of Kaplan-Meier) curves are shown in the Figure. The unadjusted HR for TKA in the arthroscopic surgery group compared with the control group was 0.80 (95% CI, 0.50-1.30) (Table 3). After adjusting for covariates, the HR was 0.85 (95% CI, 0.52-1.40) (Table 3). Greater KL grade was the only variable associated with TKA (HR, 2.06 [95% CI, 1.21-3.53]) (Table 3). Results were similar for the secondary outcome of TKA or osteotomy on either knee (adjusted HR, 0.91 [95% CI, 0.59-1.41]) (Figure, B, and Table 3). There was no violation in the proportional hazards assumption for the exposure when assessed with either the time-interaction or Schoenfeld residuals.

**Figure.**
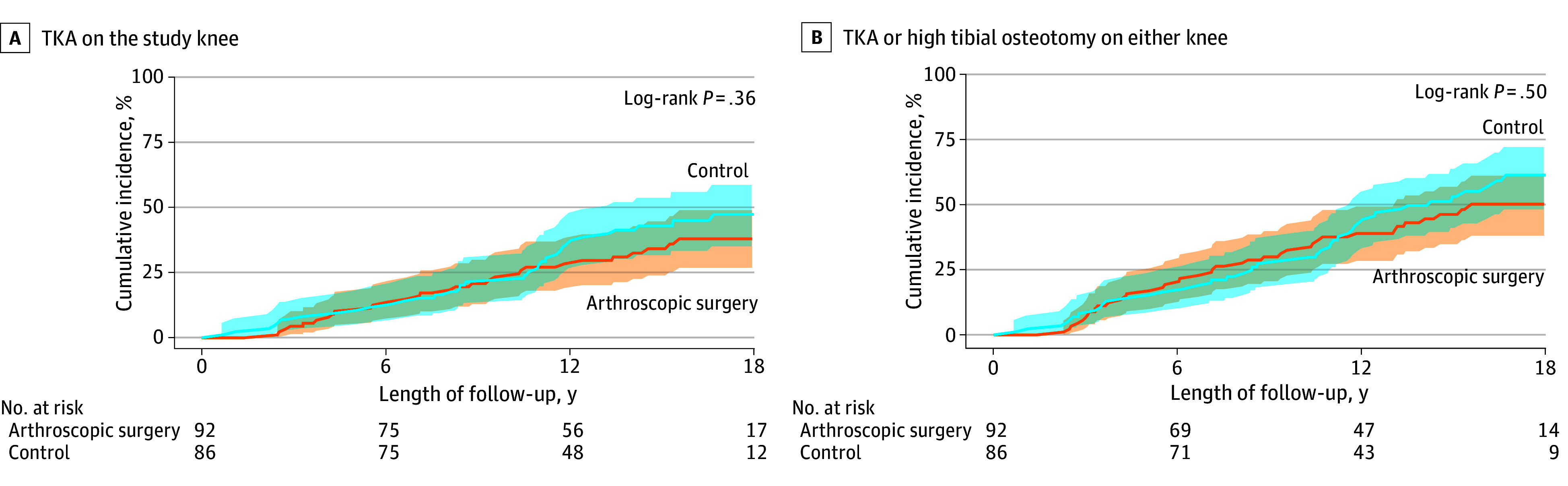
Time to Event Curve for Primary and Secondary Outcomes Kaplan-Meier estimates and cumulative incidence of the primary end point of total knee arthroplasty (TKA) on the study knee (A) and the secondary end point of total knee arthroplasty or high tibial osteotomy on either knee (B) are shown for patients randomly assigned to knee arthroscopic surgery plus nonoperative care (n = 92) or nonoperative care only (control) (n = 86).

**Table 3.** Cox Proportional Hazards Regression Model for the Primary and Secondary Outcomes for all 178 Patients^a^

Variable	Reference category	HR (95% CI)
**TKA on study knee (primary outcome)**		
Unadjusted		
Arthroscopic surgery	Control	0.80 (0.50-1.30)
Adjusted		
Arthroscopic surgery	Control	0.85 (0.52-1.40)
Age per 10 y	NA	1.16 (0.89-1.50)
Female sex	Male sex	1.41 (0.80-2.46)
Body mass index per 5 U^b^	NA	1.07 (0.89-1.29)
Kellgren and Lawrence grade >2	Kellgren and Lawrence grade 2	2.06 (1.21-3.53)
Baseline WOMAC score per 200 points^c^	NA	1.01 (0.91-1.12)
**TKA or high tibial osteotomy on either knee (secondary outcome)**		
Unadjusted		
Arthroscopic surgery	Control	0.87 (0.57-1.32)
Adjusted		
Arthroscopic surgery	Control	0.91 (0.59-1.41)
Age per 10 y	NA	1.10 (0.87-1.39)
Female sex	Male sex	1.34 (0.82-2.19)
Body mass index per 5 U^b^	NA	1.03 (0.87-1.22)
Kellgren and Lawrence grade >2	Kellgren and Lawrence grade 2	1.91 (1.21-3.01)
Baseline WOMAC score per 200 points^c^	NA	1.02 (0.93-1.11)

Abbreviations: HR, hazard ratio; NA, not applicable; TKA, total knee arthroplasty; WOMAC, Western Ontario and McMaster Universities Osteoarthritis Index.

^a^ We conducted a complete case analysis in which 5 or fewer participants were excluded due to missing covariates (ie, body mass index, Kellgren and Lawrence grade, and/or baseline WOMAC score).

^b^ Calculated as weight in kilograms divided by height in meters squared.

^c^ Includes 3 subscales (pain, stiffness, and physical function) calculated from 24 questions. Values range from 0 (no disease) to 2400 (more severe disease).

The time-stratified analysis had a consistent interpretation (Table 4): Within 5 years, the cumulative incidence was 10.2% vs 9.3% in the arthroscopic surgery group and control group, respectively (time-stratified HR for 0-5 years, 1.06 [95% CI, 0.41-2.75]); within 10 years, the cumulative incidence was 23.3% vs 21.4%, respectively (time-stratified HR for 5-10 years, 1.06 [95% CI, 0.45-2.51]). The time-stratified HR for greater than 10 years of follow up was 0.62 (95% CI, 0.29-1.35).

**Table 4.** Cox Proportional Hazards Regression Model Using the Heaviside Function on Primary and Secondary Analyses^a^

Variable	Reference category	Adjusted HR (95% CI)	
		TKA on the study knee (n = 178)	TKA or high tibial osteotomy on either knee (n = 178)
Arthroscopic surgery^b^			
For <5 y	Control	1.06 (0.41-2.75)	1.11 (0.51-2.41)
For 5 to 10 y	Control	1.06 (0.45-2.51)	1.19 (0.55-2.59)
For >10 y	Control	0.62 (0.29-1.35)	0.62 (0.31-1.27)
Age (per 10 y)	NA	1.15 (0.89-1.49)	1.09 (0.87-1.38)
Female sex	Male sex	1.40 (0.80-2.45)	1.33 (0.82-2.18)
Body mass index per 5 U^c^	NA	1.07 (0.89-1.29)	1.03 (0.87-1.22)
Kellgren and Lawrence grade >2	Kellgren and Lawrence grade 2	2.08 (1.21-3.56)	1.92 (1.21-3.04)
Baseline WOMAC score per 200 points^d^	NA	1.01 (0.91-1.12)	1.01 (0.93-1.11)

Abbreviations: HR, hazard ratio; NA, not applicable; TKA, total knee arthroplasty; WOMAC, Western Ontario and McMaster Universities Osteoarthritis Index.

^a^ We conducted a complete case analysis in which 5 or fewer participants were excluded due to missing covariates (ie, body mass index, Kellgren and Lawrence grade, and/or baseline WOMAC score). For TKA on the study knee as the outcome, 13 of 86 patients in the control group had a scope during long-term follow-up. For TKA or high tibial osteotomy on either knee as the outcome, 12 of 86 patients in the control group had a scope in follow-up. Five or fewer participants who switched from the control group to knee arthroscopy (exposure) had missing laterality, which was coded as same knee as study.

^b^ Time-stratified extended Cox proportional hazards regression model (Heaviside functions) with adjusted HR (95% CI) at different periods since randomization.

^c^ Calculated as weight in kilograms divided by height in meters squared.

^d^ Includes 3 subscales (pain, stiffness, and physical function) calculated from 24 questions. Values range from 0 (no disease) to 2400 (more severe disease).

Results of the additional analyses are presented in eTables 5 to 8 in Supplement 2. Thirteen of the 86 patients (15.1%) in the control group received knee arthroscopic surgery during the long-term follow-up. When these crossovers were included as a time-varying covariate, the association between treatment group and TKA remained consistent with the primary analysis for both primary (HR, 0.88 [95% CI, 0.53-1.44]) and secondary (HR, 1.08 [95% CI, 0.69-1.68]) outcomes (eTable 5 in Supplement 2). Results were also similar in the as-treated analysis and when evaluating only patients with KL grade 2 or when evaluating only patients with symptoms of knee catching or locking (eTables 6-8 in Supplement 2).

## Discussion

In this long-term follow-up (median, 13.8 years) of a randomized clinical trial, the cumulative incidence of TKA was similar between patients with OA of the knee assigned to nonoperative management plus arthroscopic surgery (31 [33.7%]) compared with nonoperative management only (36 [41.9%]). For both treatment groups, approximately 10% of patients at 5 years and 20% of patients at 10 years underwent TKA, which is similar to the estimated annual incidence of 2.46% after arthroscopic surgery for OA of the knee.^67^ Our study findings do not support the use of arthroscopic surgery for OA of the knee. The present results complement previously published results from this clinical trial suggesting arthroscopic surgery does not provide additional benefit to nonoperative management for improving pain, stiffness, and function^8^ and is likely not cost-effective^53^ at 2 years of follow-up. The present long-term follow-up, suggesting neither a delay nor a hastening of TKA, is relevant to patients with OA of the knee who may be considering knee arthroscopic resection of degenerative knee tissues and to those patients who have previously undergone that procedure.

We are not aware of similar long-term follow-up of randomized clinical trials investigating the incidence of TKA after arthroscopic surgery for OA of the knee. The Meniscal Tear in Osteoarthritis Research (METEOR) trial completed a 5-year follow-up among patients with OA of the knee randomized to arthroscopic partial meniscectomy vs physical therapy.^65^ Although not statistically significant, their findings suggested an indication toward greater risk of TKA in patients randomized to arthroscopic partial meniscectomy based on the intention-to-treat analysis (HR, 2.0 [95% CI, 0.8-4.9]), with the point estimate increasing in the as-treated analysis (HR, 4.9 [95% CI, 1.1-20.9]).^65^ Eligibility criteria for the METEOR trial required participants to have OA of the knee with evidence of a meniscal tear on magnetic resonance imaging.^9^ Also, the trial focused on arthroscopic partial meniscectomy vs a physical therapy regimen with the option to cross over to surgery. Compared with the present study, participants in the METEOR trial^65^ may represent a different clinical phenotype of patients with OA of the knee and degenerative meniscal tears who underwent somewhat different arthroscopic surgical and nonoperative treatments. The indication toward greater risk of TKA after arthroscopic surgery may also be due to residual confounding (as acknowledged by those authors), as a substantial number of patients crossed over (38%) or declined continued participation (19%) over the 5-year follow-up.^65^

Data from 2 multicenter longitudinal cohort studies among patients with or at risk of developing OA of the knee^23,24^ have suggested a greater risk of TKA in patients reporting a history of knee arthroscopic surgery. Using propensity score matching, Rongen et al^23^ found an HR of 3.03 (95% CI, 1.67-5.26) at 9 years of follow-up among participants in the Osteoarthritis Initiative (OAI). Using prediction models, Liu et al^24^ found an adjusted HR of 1.26 (95% CI, 1.15-1.38) at 5 years of follow-up among participants in the OAI and 1.99 (95% CI, 1.52-2.61) among participants in the Multicenter Osteoarthritis Study. Uncertainty surrounding the arthroscopic procedures performed and their indications may explain differences with the present results. Also, residual confounding, and particularly confounding by indication, is an important limitation of observational studies that may overestimate causal effects.^51^ In these observational studies, it is plausible that the same factors that led to the knee arthroscopic surgery (including unmeasured features of OA severity, access to surgical vs nonoperative care, willingness to undergo surgery, etc) also led to the greater incidence of TKA among those patients.

While we acknowledge the potential differences between studies in their participant characteristics and the arthroscopic procedures performed (eg, the type or size of meniscal tear and/or the amount of tissue resected), 2 rigorous longitudinal cohort studies,^23,24^ 1 previously reported randomized clinical trial,^65^ and the present randomized clinical trial^8^ all indicate that arthroscopic surgery for patients with OA of the knee does not delay TKA. Notably, the intention-to-treat analysis for the 2 randomized trials^8,65^ (ie, preserving the balance of prognostic factors between treatment groups due to randomization) also did not detect a statistically significant increase in risk of TKA.

### Strengths and Limitations

Strengths of this study include up to 20-year prospective collection of end points in a randomized clinical trial by linkage to high-quality administrative health data, providing an example of the unique characteristics enabled by this type of design.^55,56,68^ Crossovers were limited (15.1%) and accounted for using robust analyses. Participants randomized to either treatment arm were treated by the same health care team at a single orthopedic center in a single health system, providing similar access to operative and nonoperative treatments. This supports the internal validity of the study design throughout the extended follow-up. The sample is representative of the target population of patients with OA of the knee who still commonly undergo the arthroscopic procedures investigated.^5,6,29,34,35,36,37,38,39^ Patients in this study who underwent arthroscopic surgery had degenerative meniscal tears (>80%) and articular cartilage fragments (>95%) that were partially resected or debrided, and those procedures are consistent with the current, commonly used coding procedures for knee arthroscopic surgery.^6,10,38,69^

Our study also has important limitations. It was powered to detect differences in 2-year patient-reported outcomes rather than long-term incidence of TKA.^8^ Effects should be interpreted considering the estimated confidence intervals, which include a possible increase or decrease in incidence, albeit of smaller effect size than suggested by most previous studies.^23,29,65^ This limitation is partially mitigated by the duration of follow-up that enabled the substantial number of events (TKA) and similar or better precision than most previous studies on the topic.^23,24,29,65^ Also, although clearly important for patients, clinicians, and policy makers, the present study end points are ultimately decisions made by patients after consultation with clinicians. The reasons and opportunities to undergo TKA vary considerably, and the granularity of the clinical decision-making to proceed to TKA is lacking in our study. Like the aforementioned studies, the decision to undergo TKA or osteotomy may have been influenced by personal and health system factors, including nonoperative treatments, not measured in the present study. However, the similar long-term incidence suggests those confounders did not differ substantially between the present randomly assigned treatment groups.

## Conclusions

This secondary analysis of a randomized clinical trial of arthroscopic surgery for patients with OA of the knee did not identify a statistically significant effect on the long-term incidence of TKA. These findings suggest the addition of arthroscopic surgery to nonoperative management for patients with OA of the knee did not delay or hasten TKA. Approximately 80% of patients did not undergo TKA within 10 years of nonoperative management with or without additional knee arthroscopic surgery.

## Notes

Supplement 1.
Trial Protocol and Statistical Analysis Plan

Supplement 2. **eFigure.** Patient Flow Diagram **eTable 1.** Description of Ontario Health Administrative Databases **eTable 2.** Variables **eTable 3.** Full Baseline Demographic and Clinical Characteristics **eTable 4.** Use of Medical, Physical, and Surgical Therapy in the Patients **eTable 5.** Cox Proportional Hazards Regression Model for Primary and Secondary Outcomes, With Knee Arthroscopy During Long-Term Follow-Up (Crossovers) Used as a Time-Varying Covariate **eTable 6.** Cox Proportional Hazards Regression Model for Primary and Secondary Outcomes, With Knee Arthroscopy During Long-Term Follow-Up for the As-Treated Analysis **eTable 7.** Cox Proportional Hazards Regression Model for Primary and Secondary Outcomes in Patients With a Kellgren and Lawrence Grade of 2 (n = 78) **eTable 8.** Cox Proportional Hazards Regression Model for Primary and Secondary Outcomes in Patients Experiencing Knee Catching or Locking (n = 87)

Supplement 3.
Data Sharing Statement
