# Supplementary material for: Incidence of Total Knee Arthroplasty After Arthroscopic Surgery for Knee Osteoarthritis: A Secondary Analysis of a Randomized Clinical Trial
Source: JAMA Netw Open. 2024 Apr 18;7(4):e246578. doi: 10.1001/jamanetworkopen.2024.6578 (PMC12503430; doi:10.1001/jamanetworkopen.2024.6578)
Supplement: Supplement 1. — Trial Protocol and Statistical Analysis Plan [file jamanetwopen-e246578-s001.pdf]

STUDY PROTOCOL

Table of Contents

Original Trial Protocol...p. 2

ICES Western Dataset Creation and Statistical Analysis Plan..p. 12

A Randomized Trial of Arthroscopic Surgery for Osteoarthritis of the Knee

Background

Osteoarthritis (OA) of the knee is a degenerative disease that causes joint pain, stiffness, and decreased function. Treatment is multidisciplinary and may involve physical therapy, medication, and surgery. Arthroscopic procedures include debridement (smoothing articular surfaces), partial resection of the meniscus, and removing loose bodies and/or osteophytes. Although arthroscopic surgery is widely used to treat OA of the knee, scientific evidence to support its efficacy is lacking, and its value is highly debated.

Objectives

The primary objective is to determine the effectiveness of arthroscopic surgery in addition to optimized physical and medical therapy compared to optimized physical and medical therapy alone in patients with OA of the knee.

A secondary objective is to determine the cost-effectiveness of arthroscopic surgery in addition to optimized physical and medical therapy compared to optimized physical and medical therapy alone.

Methods

Trial Design

We will conduct an evaluator blinded, phase III, single-center, randomized controlled trial in patients with osteoarthritis of the knee.

Interventions:

Group 1: Non-operative treatment

Physical Therapy

All patients will be provided with a prescription for outpatient physical therapy services. Those patients assigned to the non-surgical treatment group will begin physical therapy as soon as possible after the study start date. Physical therapy services will be provided by hospital outpatient physical therapy departments or private practice clinicians in the patient's community. Outpatient physical therapy services will be provided at a frequency of approximately one treatment session per week, for 12 consecutive weeks. Treatment sessions are approximately one hour in duration. A structured program was developed based on a review of the literature[1-3] and a formal survey of the Canadian University physiotherapists with an interest in the treatment of patients with OA. This program is used as a guide, however, as therapists are allowed to modify intensity, order, frequency etc. at their discretion based on the individual needs of the patients. We have purposely designed the study to not have physical therapy provided by a single therapist or one department as this would significantly affect the generalizability of the study results. Rather, we have selected what we feel is the optimal balance between rigid standardization and the "real world" treatment by having a well-designed therapy guide used as a basis for treatments by many therapists.

The home exercise program is presented in two exercise booklets, which will be provided to each patient and the involved physical therapist. Stage I emphasizes range of motion and Stage II emphasizes strengthening exercises. Because patients vary in severity of OA as well as age and their particular needs, the specific exercises that each patient emphasizes and their progression through the exercises will vary and will be determined by the treating physical therapist. As well as exercises, physical therapy services will include instruction in performing activities of daily living, walking, stairs, use of cold and heat modalities, and instruction for a home exercise program. The home exercise program will be based on the booklets and requires 15 min to 20 min to complete on each occasion. Patients will be asked to complete the home exercises two times a day, and once on the days that they attend physical therapy where the involved therapist will review the exercises. If the patients cannot complete the designated exercises in 20 minutes or

less, they will stop at that point and resume exercises in the second exercise session. Patients will be advised to decrease their exercise workload if pain or swelling develops. Once patients have completed the 12 weeks of supervision they will continue unsupervised with the home exercise program for the duration of the study.

Compliance will be assessed by recording missed physical therapy treatment appointments (by the therapist) and by evaluating patient exercise diaries.

Outpatient physical therapists that have questions regarding the exercise programs will have contact information with a member of Musculoskeletal and Rehabilitation Services at our institution.

Education
For the education portion of the study, we have made arrangements with the local Arthritis Society for our study patients to be included in their workshops. Those patients who cannot attend during the day will be offered a workshop to be held as a one day session on the weekend. In addition, the research nurse who is blinded to group assignment will also conduct education sessions outlining: pathophysiology of osteoarthritis, benefits of a walking program, benefits of weight reduction, proper joint protection principles, concepts of self-efficacy and self management, relaxation techniques and methods for solving problems that arise from illness. In this way, all of the patients are subjected to education about the disease, either from the research nurse alone or from both the nurse and the Arthritis Society.

Participants will receive a copy of "The Arthritis Helpbook"[4]. A 10 minute educational video-tape that outlines these concepts will be available on a loan basis to patients to act as review.

Medical Management
Most participants will have progressed through a variety of medications for pain control prior to their referral to the Orthopaedic Clinic. The dose and regimen of the current NSAIDs will be reviewed by the study rheumatologist and optimized. If contraindications are present for the use of NSAIDs, the patient will be prescribed acetaminophen instead. Patients will be given the opportunity to discuss their medication regimens and any adverse reactions through telephone contact with the research nurse. The rheumatologist will be made aware of these concerns and appropriate dosage/medication changes will be made. At each follow-up appointment, the research nurse will discuss the various treatment options available to the patient according to the Treatment Algorithm for OA of the Knee.

In addition, the research nurse and the principal investigator will meet after each patient's 3 month appointment to discuss their treatment. At any time during the course of the study, the research nurse may request an appointment with the patient's Orthopaedic Surgeon to further optimize their treatment.

<u>Group 2: Surgical Treatment</u>
Patients with OA of the knee have varying degrees of intra-articular derangement that may include not only degeneration of articular cartilage but also synovitis, degenerative tearing of menisci, osteophyte formation, loose bodies and degenerative tearing of ligaments. The goals of arthroscopic treatment in general are to relieve mechanical symptoms of catching, locking and pain through debridement of synovitis, loose bodies, or torn fragments of ligament, meniscus and cartilage, and impinging osteophytes.

Arthroscopy will be performed within 6 weeks of treatment assignment as a day surgery procedure with patients typically discharged 2-4 hours post-surgery. The procedure will be performed under general anaesthesia with a tourniquet and thigh holder. An arthroscopic evaluation of all 3 compartments will be videotaped at the start and at the end of the arthroscopic intervention. This information will be used to monitor surgical competency and potentially for subgroup analyses at study completion. One or more of the following interventions will be performed under arthroscopic guidance: 1) debridement of degenerative tears

of menisci, 2) excision of loose articular cartilage fragments, 3) debridement of osteophytes that prevent full extension, 4) synovectomy, 5) irrigation of medial, lateral and patellofemoral joint compartments with at least one litre of saline (lavage). Abrasion or microfracture of chondral defects will not be performed.

Those patients assigned to the arthroscopic surgery group will start physiotherapy within 7 days of their surgery following an identical program to the non-surgical group.

Randomization

Each of four orthopaedic surgeons will complete the necessary clinical and radiographic assessments to establish the diagnosis of OA. The principal investigator and the research coordinator will review and confirm these assessments and establish the disease severity based on the Altman[2] and the modified Kellgren and Lawrence classifications.[5] Disagreements will be settled by consensus. Although the inter- and intra-observer reliability for radiographic classification of OA is good,[60] the correlation between radiographs and arthroscopy is only moderate with radiographs consistently underestimating the severity of articular cartilage loss.[5,-7] For the purpose of evaluation of progression of disease, radiographs are clearly an insensitive measure.[8] However, for the purposes of stratification (ensuring equal distribution of disease severity between the two treatment groups) plain radiographs are easily attainable, inexpensive and effective.

After obtaining informed consent, patients will be randomized to the non-surgical group (medical management, physical therapy, and health education) or to the surgical plus non-surgical treatment group (arthroscopy with lavage and debridement in conjunction with the same treatment as the non-surgical group). A computer-generated, stratified blocked randomization procedure will be used to guarantee that at no time during the course of the trial will the imbalances be large. Randomization will be stratified by surgeon (4 arthroscopists) and by disease severity (grades II, III, IV). Both factors are considered to be strong predictors of patient outcome. As this is not a blinded trial at the surgical level, the block size will be varied (either 2 or 4) to avoid revealing the last patient assignment in the treatment arm. The size of the block will be determined at random for each surgeon by disease severity stratum. Randomization will be done centrally, by telephone, from the data management centre (Robarts Clinical Trials).

Eligibility

We will recruit patients presenting to the Fowler Kennedy Sport Medicine Clinic, University of Western Ontario, London, Ontario who have been referred from family physicians, rheumatologists or other specialists due to unresolved symptoms from OA of the knee. All of the patients referred to this clinic have already received some form of non-surgical treatment although the type, duration and quality are variable.

Inclusion Criteria

- Idiopathic or secondary osteoarthritis of the knee as diagnosed by Altman et al[9] classification tree (83% sensitivity, 93% specificity)
- Grades II to IV severity of OA by radiographic evaluation. (modified Kellgren & Lawrence grading system)[5]
- Age greater than 18.

Exclusion Criteria

- Inflammatory or post-infection arthritis of the knee
- Previous arthroscopy for treatment of knee OA
- Isolated medial compartment OA Grade III-IV with >5^0 mechanical varus (ideal candidate for high tibial osteotomy)
- Isolated lateral compartment OA Grade III-IV with >5% mechanical valgus (ideal candidate for high tibial osteotomy or distal femoral osteotomy)
- Grade IV OA in 2 compartments and >60 years of age (ideal candidate for total knee arthroplasty)

- Cortico-steroid injection within the last three months.
- Major neurological deficit
- Major medical illness (life expectancy < 2 years or with unacceptably high operative risk).
- Pregnant
- Unable to speak or read English
- Psychiatric illness that limits informed consent
- Unlikely to comply with follow-up

Outcome Measures
The primary outcome measure is the Western Ontario McMaster osteoarthritis index (WOMAC) at the 2 year follow-up. The WOMAC is the most sensitive tool for assessing disease-specific quality of life for patients with osteoarthritis of the hip and knee.[10] It consists of three dimensions (pain [5 questions], stiffness [2 questions], and physical function [17 questions]). The measure is reproducible and has been shown to be sensitive to small but clinically important changes in clinical trials assessing NSAID's and surgical treatment.[11]

Secondary outcomes will include: the SF-36, the MACTAR questionnaire and the Arthritis Self-Efficacy scale. A utility score will be generated using the Standard Gamble technique for the purpose of the cost-effectiveness analysis. Each patient will also complete a cost diary to report any additional healthcare resource use and time off of paid employment due to the study knee.

Participants will also be asked to record the frequency of home exercises and physical therapy sessions in a compliance diary.

Mortality and morbidity associated with arthroscopic surgery and/or rehabilitation are rare, but in the event that a participant dies or suffers serious complications, this data will be collected and compared between groups. Data on any further knee surgery during the 2 year follow-up will also be collected.

Follow-up
Patient demographics and disease variables that have been suggested in previous studies to correlate moderately with outcome after arthroscopic surgery for OA of the knee (age, sex, mechanical symptoms, disease severity, pain type, duration of symptoms)[12-18] will be collected at baseline. In addition, patients will also complete the WOMAC, SF-36, MACTAR, Arthritis Self-efficacy scale, and Standard Gamble questionnaires. All outcomes will be measured at each follow-up visit (3, 6, 12, 18 and 24 months) after the initiation of treatment.

Although the primary outcome is at the 2 year point it would be extremely valuable to know if arthroscopic surgical treatment provides shorter term improvement in the outcome measures and at what point the disability imparted by the surgical procedure returns to baseline. In addition, it is our experience that compliance with treatment and drop-out rate are both highly correlated with intensity of follow-up. If patients are enrolled and followed-up only yearly, then the compliance will likely be poor (and difficult to monitor) and the drop-out rate will be unacceptably high. All outcome measures will be assessed at each follow-up appointment. In addition physical therapy compliance diaries and costing diaries will be reviewed. Participants will also be contacted by the study coordinator on a monthly basis by telephone to encourage compliance with home exercise, weight loss, and medication use.

Sample Size
The primary outcome will be the WOMAC Osteoarthritis Index at 2 year follow-up. A group sequential design[19] will be adopted for this trial, with a maximum sample size of 186 patients.

The sample size considerations were based upon the following parameters and assumptions:

1) The difference in mean WOMAC scores at 2 years between the two groups was estimated to be 207 points (i.e., a 20% reduction from the non-surgical group), and a standard deviation of 452 points. These figures were extrapolated from 40 patients in the control arm of a study of orthopaedic braces carried out at our institution.[20] The baseline WOMAC score was 940, which increased to 964 at 6 months. Given that osteoarthritis is generally a slow, progressive disease, it was predicted that the WOMAC score would increase by 24 units every 6 months, thus yielding a score of 1036 at 2 years. A 20% reduction in the WOMAC score is considered by surgeons to be minimally clinically important.
2) The sample size formula for comparing two independent group means was used.
3) A two-tailed alpha of 5% and power of 80% was used.
4) An O'Brien-Fleming boundary was used for the stopping rules.
5) The sample size has been adjusted upwards to account for a 15% drop-out rate.

All calculations were done using the computer software program EaSt.[21]

Plan for Statistical Analysis

Primary Analyses

The primary analysis will be based upon the intention-to-treat principle, whereby all patient outcomes will be attributed to the assigned treatment arm. The WOMAC score at two years will be compared between groups adjusting for baseline scores. The MACTAR Questionnaire, Arthritis Self-Efficacy Scale, and SF-36 data will be analyzed in a similar manner. All tests will be carried out at a 5% significance level. No p-value adjustment will be required for multiple outcomes, as treatment effectiveness will only be based upon the primary variable (i.e.,WOMAC score). The other three outcome variables are secondary and have been pre-specified at the outset of the trial.

Economic Analysis

Economic evaluation compares alternative health care technologies in terms of their costs and consequences. Incremental analysis performed from the perspectives of the health-care system, the patient/family and society will compare arthroscopic surgery and usual medical care for between-group differences over time in patient outcomes and economic net-costs (costs less benefits). Cost-benefit, cost-effective and cost-utility analyses will be performed. Future costs and consequences will be adjusted for differential timing using the 5% discount rate. Sensitivity analyses will be performed to examine the effects of assumptions and for uncertainty surrounding estimated between-group differences.

The direct costs of arthroscopic surgery include pre-operative diagnostic testing and consultation, the use of the operating room, equipment, caregiver time (surgeon, anaesthetist, nurses and other staff) plus post-operative care in the recovery room and surgical nursing unit. Our institution is fully compliant with the Management Information System (MIS) Guidelines, therefore the fully-allocated costs of all care and services utilized during each inpatient episode will be available for all patients. The direct costs borne by family members will also be estimated as the time lost from work by caregivers, valued using appropriate average wages, and the distances travelled, valued using representative charges for automobile-related expenses. The indirect costs of the lost production of employed patients because of hospitalization will also be estimated using appropriate wage rates.

Important direct costs during follow-up include the utilization of ambulatory (e.g., hospital, community physiotherapy, physicians), inpatient (University Hospital and elsewhere), community (e.g., home care) and patient/family (time and travel) resources as well as the consumption of medications. Follow up costs will be collected using both the patient diary and questioning during follow-up appointments. For utilization related to patients' OA condition, the per diem costs of services provided in ambulatory and inpatient settings, OHIP

tariffs for physician visits and the Ontario Formulary prices of medications will be used to estimate follow-up costs. The direct costs borne the patient/family and the indirect benefits derived from changes in employment status will be calculated as above.

Trial Management

The study will be monitored for safety and effectiveness. The monitoring will be done by an external committee in conjunction with the data management center. The committee will serve strictly in an advisory capacity and will not be involved in conducting the trial. The monitoring committee will be blinded to the patients' treatment assignment. Group assignments will be designated as A and B. At each effectiveness review, the WOMAC scores will be compared between the two groups to decide whether the study should be stopped for either substantial effectiveness or futility. This approach will ensure an overall alpha of 5% for the study. The rationale for using a group sequential design instead of waiting for the end of the follow-up period is that such a design might make it possible to report study findings earlier. It is emphasized that the group sequential boundaries are not absolute rules in stopping this trial early. The monitoring committee will examine all the data and consider other factors, such as consistency of results across the trial's duration, across secondary measures of effectiveness, and across surgeons. One final data analysis will be conducted at the conclusion of the trial when 100% of the recruited patients have reached 2 years follow-up.

The study coordinator is responsible for the day to day operations of the trial. Because this is a “surgery vs no surgery” study there are considerable complexities involved in maintaining blinding. The coordinator’s duties include booking surgery, randomization, answering questions about surgery prior to surgery, monthly telephone support calls, checking of data forms prior to submission to the Robarts Clinical Trials, and acting as the liaison with the data management group responding to all queries. The coordinator also is responsible for organizing the investigator meetings, taking minutes at these meetings, writing reports and will be involved in the organization of the meetings for data review, abstract, manuscript, and presentation preparation. The coordinator will report to the Steering Committee.

Executive Committee

The Executive Committee will consist of Dr. Brian Feagan, and Dr. Peter Fowler. They will meet every 3 months throughout the duration of the trial. This committee will be responsible for overseeing the management of the trial. They will ensure that the protocol is adhered to, data is appropriately collected and managed and they will oversee the analysis and manuscript preparation. The Safety Monitoring Committee will report to the Executive Committee.

Steering Committee

The Steering Committee will consist of Dr. Peter Fowler, the study coordinator, the study nurse, and the Robarts Clinical Trials staff member coordinating the data management. They will meet on a weekly basis throughout the study. This committee will be responsible for overseeing the day to day operations of the trial.

Safety Monitoring Committee

This committee will consist of two people who are not investigators in the trial. Dr. Alan Donner is the Chair of Epidemiology and Biostatistics at the University of Western Ontario. He is an independent investigator familiar with the methodology of rigorous randomized clinical trials and the safety and ethical issues related to the treatment of patients with OA of the knee. The other member is Dr. Daniel Whelan, an orthopaedic surgeon with epidemiology training. This committee will review the safety data and make recommendations to the Executive Committee.

References

1. Fisher, N. M., Gresham, G. E., Abrams, M., Hicks, J., Horrigan, D., and Pendergast, D. R. Quantitative effects of physical therapy on muscular and functional performance in subjects with osteoarthritis of the knees. Arch.Phys Med Rehabil;74(8):840 - 847, 1993.

2. Fisher, N. M., Pendergast, D. R., Gresham, G. E., and Calkins, E. Muscle rehabilitation: its effect on muscular and functional performance of patients with knee osteoarthritis. Arch Phys Med Rehabil;72(6):367 - 374, 1991.

3. Jackson, R. W. and Rouse, D. W. The results of partial arthroscopic meniscectomy in patients over 40 years of age. J Bone Joint Surg Br.;64(4):481 - 485, 1982.

4. Lorig K, Fries J. The arthritis helpbook: a tested self-management program for coping with arthritis and fibromyalgia. 5th ed. New York: Persues books; 2000.

5. Blackburn, W. D., Jr., Bernreuter, W. K., Rominger, M., and Loose, L. L. Arthroscopic evaluation of knee articular cartilage: a comparison with plain radiographs and magnetic resonance imaging. J Rheumatol.;21(4):675 - 679, 1994.

6. Brandt, K. D, Fife, R. S., Braunstein, E. M., and Katz, B. Radiographic grading of the severity of knee osteoarthritis: relation of the Kellgren and Lawrence grade to a grade based on joint space narrowing, and correlation with arthroscopic evidence of articular cartilage degeneration. Arthritis Rheum.;34(11):1381 - 1386, 1991.

7. Fife, R. S., Braunstein, E. M., Katz, B., Shelbourne, K. D., Kalasinski, L. A., and Ryan, S. Relationship between arthroscopic evidence of cartilage damage and radiographic evidence of joint space narrowing in early osteoarthritis of the knee. Arthritis Rheum;34(4):377 - 382, 1991.

8. Altman, R., Fries, J. F., Bloch, D., Carstens, J., Cooke, T. D., Genant, H., Gofton, P., Groth, H., McShane, D. J., and Murphy, W. Radiographic assessment of progression in osteoarthritis. Arthritis Rheum.;30(11):1214 - 1225, 1987.

9. Altman, R., Asch, E., Bloch, D., Bole, G., Borenstein, D., Christy, W., Cooke, T. D., Greenwald, R., and Hochberg, M. Development of criteria for the classification and reporting of osteoarthritis. Classification of osteoarthritis of the knee. Diagnostic and Therapeutic Criteria Committee of the American Rheumatism Association. Arthritis Rheum.;29(8):1039 - 1049, 1986.

10. Bellamy, N. WOMAC Osteoarthritis Index: A User's Guide. 1995.

11. Laupacis, A., Bourne, R., Rorabeck, C., Feeny, D., Wong, C., Tugwell, P., Leslie, K., and Bullas, R. Costs of elective total hip arthroplasty during the first year. Cemented versus noncemented. The Journal of Arthroplasty;9(5):481 - 487, 1994.

12. McGinty, J. B., Johnson, L. l., Jackson, R. W., McBryde, A. M., and Goodfellow, J. W. Uses and abuses of arthroscopy: a symposium. J Bone Joint Surg Am;74(10):1563 - 1577, 1992.

13. Baumgaertner, M. R., Cannon, W., Vittori, J. M., Schmidt, E., and Maurer, R. C. Arthroscopic debridement of the arthritic knee. Clin.Orthop.;(253):197 - 202, 1990.

14. Edelson, R., Burks, R., and Bloebaum, R. D. Short-term effects of knee washout for osteoarthritis. Am J Sports Med;23(3):345 - 349, 1995.

15. Novak, P. J. and Bach, B. Selection criteria for knee arthroscopy in the osteoarthritic patient. Orthop Rev;22(7):798 - 804, 1993.

16. Salisbury, R. B., Nottage, W. M., and Gardner, V. The effect of alignment on results in arthroscopic debridement of the degenerative knee. Clin.Orthop.;(198):268 - 272, 1985

17. Timoney, J. M., Kneisl, J. S., Barrack, R., and Alexander, A. H. Arthroscopy update #6. Arthroscopy in the osteoarthritic knee. Long- term follow-up. Orthop Rev;19(4):371 - 379, 1990.

18. Yang, S. S. and Nisonson, B. Arthroscopic surgery of the knee in the geriatric patient. Clin Orthop;(316):50 - 58, 1995.

19. Lan, K. K. and DeMets, D. L. Discrete sequential boundaries for clinical trials. Biometrika;70659 - 663, 1983.

20. Kirkley, A., Webster-Bogaert, S., Litchfield, R., Amendola, A., Fowler, P., MacDonald, S., and McCalden, R. The effect of bracing on medial compartment osteoarthritis of the knee. Journal of Bone and Joint Surgery;81A(4):539 - 548, 1998.

21. Cytel Software Corporation EaSt: A software package for the design and interim monitoring of group sequential clinical trials. 1998.

Appendix: Clarifications and Amendments

A) Treatment of Meniscal Tears (May 2004)

Background and Rationale: Degeneration of the meniscus is part of the pathology seen in OA of the knee. The vast majority of the menisci with degenerative findings will exhibit horizontal delamination of the meniscus in which the meniscus splits into a top and bottom leaf. There are two schools of thought on the approach to these meniscal findings. One is that they give symptoms and the meniscus is not performing normally and therefore should be debrided back to a stable rim of meniscus. The other school of thought is that the meniscus likely accounts for little of the total symptom complex and still provides an important function in the knee and therefore should be left unless torn to the point of causing a mechanical blockage in the knee. There are many studies demonstrating that the more meniscus removed from an otherwise normal knee the higher the likelihood of progression to OA.[1-4]

Clarification: From the study outset we have chosen to approach this issue of meniscal degeneration in knee OA the following way: We include patients suspected of having a degenerative meniscal tear. We exclude patients suspected of having a large (bucket handle) mechanical tear of the meniscus that resulted in an acute loss of extension. If a patient presents with a mechanical blockage to motion (so called locking) and has evidence of a bone fragment acting as a loose body in the knee, s/he is not included in the study. If a patient describes a mechanical blockage to motion but the plain x-rays do not demonstrate a loose body then the patient is evaluated with an MRI. If on the MRI the patient has typical findings of OA, including degenerative tear of the meniscus, s/he is included in the study. If on the other hand, the MRI shows a mechanical meniscal tear (flap or bucket handle tear) the patient is not included in the study.

To date there have been no patients in the non-surgical arm of the trial who have gone on to develop mechanical blockage to motion of the knee. As expected, many patients in the surgical group have had meniscal changes consistent with OA. These have been treated at the time of surgery with the approach most commonly used in North America, which is to resect as little tissue as possible but to remove portions of the

meniscus that the surgeon feels might be getting tugged on and therefore contribute to the pain in the knee. This can be accomplished by smoothing the free edge with a shaving tool or a small punch. During the study there has been no case of a bucket handle tear or meniscal repair.

1. Boyd K.T. and Myers P.T. Meniscus preservation: rationale, repair techniques and results. Knee;10(1):1 - 11, 2003.
2. Cicuttini, F., Forbes, A., Yuanyuan, W, Rush, G, and Stuckey, S. L. Rate of knee cartilage loss after partial meniscectomy. Journal of Rheumatology;29(9):1954 - 1956, 2002.
3. van Tienen, T. G., Heijkants, R. G., de Groot.J.H., Pennings A.J., Poole A.R., Veth R.P., and Buma P. Presence and mechanism of knee articular cartilage degeneration after meniscal reconstruction in dogs. Osteoarthritis Cartilage;11(1):78 - 84, 2003.
4. Verdonk, R. Meniscal Transplantation. Acta Othop Belgica;68(2):118 - 127, 2002.

B) Subgroup Analysis Plan (January 2007)

Background and Rationale: Specific subgroup analyses with directional hypotheses are not clear in the original protocol.

Clarification and Amendment: We will perform two subgroup analyses among patients hypothesized to derive greater benefit from surgery: 1) patients with less severe radiographic disease (Kellgren-Lawrence grade II), and 2) patients reporting mechanical symptoms of catching and/or locking.

C) Revised Economic Analysis Plan (January 2013)

Background and Rationale: The economic analysis plan in the original protocol lacks detail.

Amendment: We will conduct a cost-effectiveness analysis from both the Canadian healthcare payer and societal perspectives. The payer perspective includes direct costs covered by the publicly funded system, including hospital, procedure-related, clinician and provider time, tests, procedures or surgeries, and medications for patients on disability or aged 65 years and older. In addition to the healthcare system costs, the societal perspective also includes any out-of-pocket costs to the patient (such as physical therapy, medication, or assistive devices not covered by the provincial insurance plan), and indirect costs such as time involved with appointments, and time off employment, homemaking or caregiving activities as a result of the intervention.

Net Benefit Regression
The net benefit regression (NBR) framework[1] provides an estimate of cost-effectiveness by considering the incremental cost and effect on an intervention in addition to the maximum acceptable willingness-to-pay (WTP) per unit of health gain; in this case, WTP refers to the amount one is willing to spend for an additional improvement on the WOMAC score, or for an additional QALY gained. An intervention is cost-effective if the incremental net benefit (INB) is greater than zero.

We will conduct two separate NBR models, the first using the WOMAC total score, and the second using QALYs as the measure of effectiveness. The WTP value was varied between $0 and $100,000. The net benefit regression method provides a means to adjust for any potentially confounding factors and therefore allows greater statistical efficiency and provides a more precise estimate of the INB.

We will include the following covariates in our models: baseline WOMAC score, baseline utility (for the QALY model), radiographic severity of knee OA (Kellgren-Lawrence grade 2, or greater than 2), age, and body mass index. Significant covariates from our regression model will also included as interaction terms.

Statistical uncertainty will be characterized using 95% confidence intervals, and visually displayed using the cost-effectiveness plane, and cost-effectiveness acceptability curves (CEAC).[2] We will use non-parametric bootstrapping to draw 1,000 ICER estimates from the original sample, and plot them on the cost-effectiveness plane. The CEAC demonstrates the probability of cost-effectiveness at various WTP values.

1. Hoch JS, Briggs AH, Willan AR. Something old, something new, something borrowed, something blue: a framework for the marriage of health econometrics and cost-effectiveness analysis. *Health Econ* 2002 Jul;11(5):415-30.
2. Hoch JS, Rockx MA, Krahn AD. Using the net benefit regression framework to construct cost-effectiveness acceptability curves: an example using data from a trial of external loop recorders versus Holter monitoring for ambulatory monitoring of "community acquired" syncope. *BMC Health Serv Res* 2006 Jun 6;6:68.

D) Future knee surgeries (December, 2018)

Background and Rationale: The original trial protocol submitted to CIHR identifies the delayed need for future more invasive surgeries (such as osteotomy and especially arthroplasty) as rationale commonly provided for the use of knee arthroscopy in patients with knee OA. Although beyond the scope of the requested funding at the time, the investigators suggested a between-group comparison of subsequent knee surgeries was an important analysis in the future. In December 2018 an initial agreement with ICES Western was established, followed by a Data Creation Plan (DCP) in , detailed below.

Study Design and Project Time Frame Definitions

**Study Design**	☒ Cohort study ☐ Matched cohort study ☐ Case-control study<br>☐ Cross-sectional study ☐ Other (specify):
**Project Timeline**	

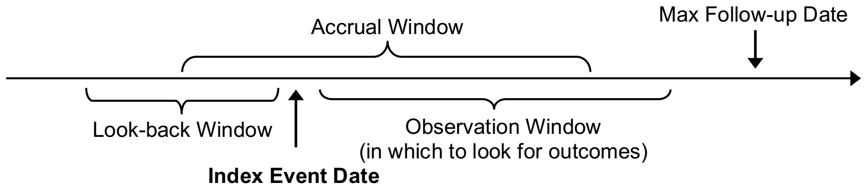

**Accrual Start/End Dates**	January 1, 1999 – August 31, 2005
**Max Follow-up Date**	March 31, 2019
**When does observation window terminate?**	Death<br>Emigration<br>Outcome date<br>Max follow-up date
**Lookback Window(s)**	Within 1 year prior to the index date

Cohort Build

Note: Include a cohort build table in appendices.

**Index Event / Inclusion Criteria and Index Date**	Patients with osteoarthritis of the knee (OA) who were referred to and assessed by an orthopedic surgeon as per the linked dataset (index date = treatment date)
**Estimated Size of Cohort (if known)**	178 patients (from previous study: A Randomized Trial of Arthroscopic Surgery for Osteoarthritis of the Knee)
**Exclusions (in order)**	

Step	Description
1	Data cleaning:<br>a) Missing or invalid IKN<br>b) Missing or invalid age (<18 or >105)<br>c) Missing or invalid sex<br>d) Death on or before the index date<br>e) Non-Ontario resident

Concept Definitions

Note: Include concept definition details in appendices.

**Main Exposure or Risk Factor**	Knee arthroscopy (KA)
**Primary Outcome Definition**	TKA on study knee
**Secondary Outcome Definition(s)**	a) TKA on study knee or osteotomy on any knee<br>b) TKA on any knee or osteotomy on any knee
**Baseline Characteristics (by timeframe, e.g. at index, in the past year, etc.)**	a) Age<br>b) Sex<br>c) Income quinitile<br>d) Rurality (rural vs. urban)<br>e) Index year<br>f) Weight (kg)<br>g) Height (cm)<br>h) BMI<br>i) Duration of OA symptoms (months)<br>j) Kellgren-Lawrence grade<br>k) Anatomical alignment (degrees)<br>l) Symptoms of catching or locking<br>m) Joint effusion<br>n) Pain with forced flexion<br>o) Tendernesss at the tibiofemoral join line<br>p) WOMAC total score (at baseline)<br>q) SF-36 physical component summary (at baseline)<br>r) ASES score (at baseline)<br>s) MACTAR score (at baseline)<br>t) Charlson comorbidity score<br>u) Previous knee arthroscopy in the past 5 years<br>v) Hospitaizations in the past year<br>w) Physician consults in the past year
**Other Concepts (if applicable)**	a) Evidence of index KA<br>b) Number of days between treatment date (RCT) and procedure date (admin data) for index KA<br>c) Evidence of agreement in study knee identification for KA<br>d) KA involving meniscus<br>e) Any KA during follow-up<br>f) Death<br>g) Emigration

Analysis Plan and Dummy Tables

Step 0: Cohort Codes

I. Review cohort code list.
II. Review linked dataset.

*****STOP FOR REVIEW*****

Step 1a: Cohort Build

I. Apply inclusion criteria.
    a. Define index date as date of treatment.
II. Obtain data for exclusions.
III. Apply exclusion 1 and track number excluded at each step in Appendix B, Tbl1 InclusionExclusion (see Appendix C, Table C1 for details).

*****STOP FOR REVIEW*****

Step 1b: Confirmation of Index Procedure

I. Obtain exposure (see Appendix C, Table C2 for details).
II. Obtain KA-related other concepts: evidence of index KA, evidence of agreement in study knee identification for KA, KA involving meniscus, and any KA during follow-up (see Appendix C, Table C4 for details).
III. Report the frequencies of each KA code for the cohort overall and by KA randomized, KA crossover, or no KA (Appendix B, Tbl2a KACodes).
IV. Report the frequencies of each requested KA-related concept for the cohort overall and by KA randomized, KA crossover, or no KA (Appendix B, Tbl2b AdminKA).

*****STOP FOR REVIEW. Determine if 8-week window to look for KA codes needs to be extended. If not all study KAs are found in code, extend window. Also, determine if there is enough data on laterality to continue analyses as written. If not, revise.*****

DECISION: Expand the window to look for KA codes to +/- 8 weeks of index date. Re-evaluate after applying this new window. Also, determine if procedure date found in admin data may be used as the index date for those with index KA procedure (KA group).

V. Create a table showing the number of days between treatment date (RCT) and procedure date (admin data) for index KA (Appendix B, Tbl2c NofDays).
VI. Exploratory: For KA patients (KA overall) with missing codes, pull most frequent OHIP feecode/CIHI-DAD/SDS CCI/CCP codes in a +/- 2 week window from index. (Appendix B, Tb|2d CodeCheck)

*****STOP FOR REVIEW.*****

DECISIONS:
1. Add additional admin codes to the index KA definition (see updated Appendix A) and expand the window to look for KA codes to +/- 12 weeks of index date.
2. Look to see how many of the individuals with missing KA alignment have NO OHIP codes in the 12 week period and/or died within that time period. Report N to team.

DECISIONS:
1. For the main analysis (ITT), include all patients based on the group to which they were randomized ("KA" or "No KA") and their treatment date from the RCT data (variable: "randomization_date").
2. For the sensitivity analysis (As Treated), the two groups for this analysis are:
    a. "Evidence of KA" group: randomized to receive KA and had a KA code in the admin data within +/- 12 weeks of the "randomization_date" variable
    b. "No evidence of KA" group: "No KA" group, plus the "KA crossover" group and the "KA randomized" patients who were missing KA codes in the admin data

   The treatment date from the RCT data (variable: "randomization_date") will be used, and any KA code in the admin data appearing 12 weeks after the treatment date or later will be included in the Cox model as a time-varying covariate.
3. Create a frequency table and report the number of KAs during follow-up and knee surgery outcomes that have laterality information available (i.e. captured with a CCI code) for the first occurence and for any occurrence for the overall cohort and by randomization group (KA vs. no KA). Also report how many of these procedures are conducted on the same knee as the study knee (Appendix B, Tbl2e Laterality).
4. Create a frequency table and report the location of scopes for the time-varying exposure (Appendix B, Tbl2f Location).

*****STOP FOR REVIEW. Decide next steps based on availability of laterality information.*****

DECISIONS:

Analysis Plan and Dummy Tables

1. Conduct a chart review to confirm: 1) KA was conducted for those who were randomized to KA but did not cross over, and 2) KA was conducted on the study knee.
2. Change primary outcome to TKA on study knee and secondary outcomes to: a) TKA on study knee or osteotomy on any knee, and b) TKA on any knee or osteotomy on any knee. Where laterality is missing for the outcome of TKA or time-varying covariate of follow-up KA, assume procedures occurred on study knee for main analyses and assume procedures occurred on alternate knee for sensitivity analyses.
3. Create a frequency table and report the location of outcome TKAs and osteotomies (Appendix B, Tbl2f Location).

Analysis Plan and Dummy Tables

Step 2: Obtain Baseline Characteristics, Outcomes, and Other Concepts

I. Obtain baseline characteristics (see Appendix C, Table C2 for details).
II. Obtain outcomes (see Appendix C, Table C3 for details).
III. Obtain remaining other concepts: death and emigration (see Appendix C, Table C4 for details).

Step 3: Descriptive Analyses

I. Create a baseline table for the cohort reporting requested information for each baseline characteristic overall and by randomization to KA or no KA (Appendix B, Tbl3 BaselineRandomization).
   a. Compute p-values using one-way ANOVA (continuous variables), chi-squared test (categorical variables), and Cochrane-Armitage test (ordinal variables), where a difference of <0.05 is considered statistically significant.
   b. Compare differences across groups using absolute standardized differences, where a difference of >0.10 is considered statistically significant.

*****STOP FOR REVIEW. Decide on which variables to adjust for in the later Cox models.*****

Analysis Plan and Dummy Tables

Step 4: Outcome Analyses (ITT, based on randomization status)

I. Create an outcome table reporting crude frequency (%), event rate, and unadjusted hazard ratio for KA and no KA groups for each outcome: TKA on study knee, TKA on study knee or osteotomy on any knee, and TKA on any knee or osteotomy on any knee. Also report the crude frequency (%) for KA and no KA groups for each censoring event: death and emigration (Appendix B, Tbl4 Outcomes).

*****STOP FOR REVIEW. Determine if the number of deaths and immigrations prior to end of follow-up are low enough to treat these as censoring events rather than competing risks. Also determine if there are enough osteotomies to necessitate all models listed below for both secondary outcomes.*****
NOTE: remove patients with missing baselines here

June 11 2021 decision: An unadjusted subdistribution was also tested and no changes were seen between the cox & subdistribution. Therfore, sticking with cox model. After observing the results above and due to risk of small cells the two remaining outcomes are TKA on study knee and TKA on any knee or osteotomy on any knee. If laterality is missing assume on same knee as study knee (TKA on study knee outcome).

II. Create Cumulative Incidence (complement of KM)curves for each outcome by randomization status (KA or no KA group): TKA on study knee, and TKA on any knee or osteotomy on any knee. If laterality information is missing for the TKA outcome, assume it occurred on the study knee. (Appendix B, Fig2 CI).

III. Run a Cox proportional hazards model for the primary outcome (TKA on study knee) by randomization status (KA or no KA group), adjusting for age, sex, bmi(continuous), KL grade (2 vs 3 and4), baseline WOMAC (continuous). Censoring events include death and emigration from Ontario. If laterality information is missing for the TKA outcome, assume it occurred on the study knee.
    a. Report aHRs, 95% CIs, and p-values (Appendix B, Tbl5 AdjustedAnalyses). Also report number of observations from adjusted model (in log output).

IV. Run a Cox proportional hazards model for the secondary outcome b) TKA on any knee or osteotomy on any knee by randomization status (KA or no KA group), adjusting for for age, sex, bmi(continuous), KL grade (2 vs 3 and4), baseline WOMAC (continuous). Censoring events include death and emigration from Ontario.
    a. Report aHRs, 95% CIs, and p-values (Appendix B, Tbl5 AdjustedAnalyses).

*****STOP FOR REVIEW.*****

Analysis Plan and Dummy Tables

Step 6: Sensitivity Analyses (As Treated)

I. Run a Cox proportional hazards model for the primary outcome (TKA on study knee) by KA or no KA group, adjusting for age, sex, bmi(continuous), KL grade (2 vs 3 and4), baseline WOMAC (continuous). Censoring events include death and emigration from Ontario. Time-varying covariate is any KA after the first 12 weeks after index date. If laterality information is missing for the follow-up KA or TKA outcome, assume they occurred on the study knee.
   a. Report aHRs, 95% CIs, and p-values (Appendix B, Tbl7 SensitivityAnalyses).

II. Run a Cox proportional hazards model for secondary outcome b) TKA on any knee or osteotomy on any knee by KA or no KA group, adjusting for age, sex, bmi(continuous), KL grade (2 vs 3 and4), baseline WOMAC (continuous). Censoring events include death and emigration from Ontario. Time-varying covariate is any KA after the first 12 weeks after index date. If laterality information is missing for the follow-up KA, assume it occurred on the study knee.
   a. Report aHRs, 95% CIs, and p-values (Appendix B, Tbl7 SensitivityAnalyses).

Post-hoc Analysis, Subgroup KL2, June 25 2021: Only subjects who had a KL grade=2, run a cox ph model adjusting for exposure and age. Note, only have enough power for 2 covariates .

1. Inclusions/exclusions: remove patients with KL grade 3 or4 (Appendix B, Tb|8).
2. Model 1: TKA on study knee (Appendix B, Tb|8a).
3. Model 2: TKA/Osteotomy on any knee (Appendix B, Tb|8b).

Post-hoc Analysis, Subgroup catching or locking, June 25 2021: Only subjects who had catching or locking=1, run a cox ph model adjusting for exposure, age, sex, KL grade (2 vs 3/4). Note, only have enough power for 4 covariates.

1. Inclusions/exclusions: remove patients with no catching or locking (Appendix B, Tb|9).
2. Cox Model 1: TKA on study knee (Appendix B, Tb|9a).
3. Cox Model 2: TKA/Osteotomy on any knee (Appendix B, Tb|9b).

New Analysis, February 22 2022:

Post-hoc Analysis, updating reference groups for covariates (Tb|5, 7, 8, 9):

In primary (ITT), secondary (ITT), sensitivity (AT), subgroup KL2, and subgroup catching or locking adjust for:

1. Exposure (KA vs No KA)
2. Age per 10 years
3. Sex (Female vs Male)
4. BMI per 5
5. KL grade (3/4 vs 2)
6. Baseline WOMAC per 200

Post-hoc Analysis, laterality:

Report number of patients with confirmed laterality and number of patients missing laterality. (Tb|10)

Post-hoc Analysis, primary (ITT) analysis details:

1. Report median (IQR) follow-up time for full cohort and by exposure (Tb|11)
2. Report CIF (%, 95% CI) at 5, 10, and 15 years of follow-up (Tb|11)
3. Add number at risk and cumulative events in CIF plot (Fig 2)

Post-hoc Analysis, secondary (ITT) analysis:

1. Add number at risk and cumulative events in CIF plot (Fig 2)

Analysis Plan and Dummy Tables

Post-hoc Analysis, sensitivity analysis 2, changing exposure:

I. Run a Cox proportional hazards model for the primary outcome (TKA on study knee) by randomization status (KA or no KA group), **of the patients who crossed over in the KA group recode them as having no KA (86 KA now and 92 no KA)**. Adjusting for age (per 10 years), sex (F vs M), bmi(continuous per 5), KL grade (3/4 vs 2), baseline WOMAC (continuous per 200). Censoring events include death and emigration from Ontario. If laterality information is missing for the TKA outcome, assume it occurred on the study knee.
   a. Report aHRs, 95% CIs, and p-values (Appendix B, Tbl12 Sensitivity Analysis 2). Also report number of observations from adjusted model (in log output).

II. Run a Cox proportional hazards model for the secondary outcome b) TKA on any knee or osteotomy on any knee randomization status (KA or no KA group), **of the patients who crossed over in the KA group recode them as having no KA (86 KA now and 92 no KA)**. Adjusting for age (per 10 years), sex (F vs M), bmi(continuous per 5), KL grade (3/4 vs 2), baseline WOMAC (continuous per 200). Censoring events include death and emigration from Ontario. If laterality information is missing for the TKA outcome, assume it occurred on the study knee.
   a. Report aHRs, 95% CIs, and p-values (Appendix B, Tb|12 Sensitivity Analysis 2). Also report number of observations from adjusted model (in log output).

New Analysis, May 27 2022:

Post-hoc Analysis, sensitivity analysis details:

I. Report median (IQR) time to KA for patients who were in No KA group but switched to KA. (Tb|13)

II. Figure 2, change to Arthroscopic Surgery and Control rather than KA and No KA
