# Supplementary material for: Incidence of Total Knee Arthroplasty After Arthroscopic Surgery for Knee Osteoarthritis: A Secondary Analysis of a Randomized Clinical Trial
Source: JAMA Netw Open. 2024 Apr 18;7(4):e246578. doi: 10.1001/jamanetworkopen.2024.6578 (PMC12503430; doi:10.1001/jamanetworkopen.2024.6578)
Supplement: Supplement 2. — eFigure. Patient Flow Diagram eTable 1. Description of Ontario Health Administrative Databases eTable 2. Variables eTable 3. Full Baseline Demographic and Clinical Characteristics eTable 4. Use of Medical, Physical, and Surgical Therapy in the Patients eTable 5. Cox Proportional Hazards Regression Model for Primary and Secondary Outcomes, With Knee Arthroscopy During Long-Term Follow-Up (Crossovers) Used as a Time-Varying Covariate eTable 6. Cox Proportional Hazards Regression Model for Primary and Secondary Outcomes, With Knee Arthroscopy During Long-Term Follow-Up for the As-Treated Analysis eTable 7. Cox Proportional Hazards Regression Model for Primary and Secondary Outcomes in Patients With a Kellgren and Lawrence Grade of 2 (n = 78) eTable 8. Cox Proportional Hazards Regression Model for Primary and Secondary Outcomes in Patients Experiencing Knee Catching or Locking (n = 87) [file jamanetwopen-e246578-s002.pdf]

Supplementary Online Content

Birmingham TB, Primeau CA, Shariff SZ, et al. Incidence of total knee arthroplasty after arthroscopic surgery for knee osteoarthritis: a secondary analysis of a randomized clinical trial. *JAMA Netw Open*. 2024;7(4):e246578. doi:10.1001/jamanetworkopen.2024.6578

eFigure. Patient Flow Diagram

eTable 1. Description of Ontario Health Administrative Databases

eTable 2. Variables

eTable 3. Full Baseline Demographic and Clinical Characteristics

eTable 4. Use of Medical, Physical, and Surgical Therapy in the Patients

eTable 5. Cox Proportional Hazards Regression Model for Primary and Secondary Outcomes, With Knee Arthroscopy During Long-Term Follow-Up (Crossovers) Used as a Time-Varying Covariate

eTable 6. Cox Proportional Hazards Regression Model for Primary and Secondary Outcomes, With Knee Arthroscopy During Long-Term Follow-Up for the As-Treated Analysis

eTable 7. Cox Proportional Hazards Regression Model for Primary and Secondary Outcomes in Patients With a Kellgren and Lawrence Grade of 2 (n = 78)

eTable 8. Cox Proportional Hazards Regression Model for Primary and Secondary Outcomes in Patients Experiencing Knee Catching or Locking (n = 87)

This supplementary material has been provided by the authors to give readers additional information about their work.

© 2024 Birmingham TB et al. *JAMA Network Open*.

eFigure. Patient Flow Diagram

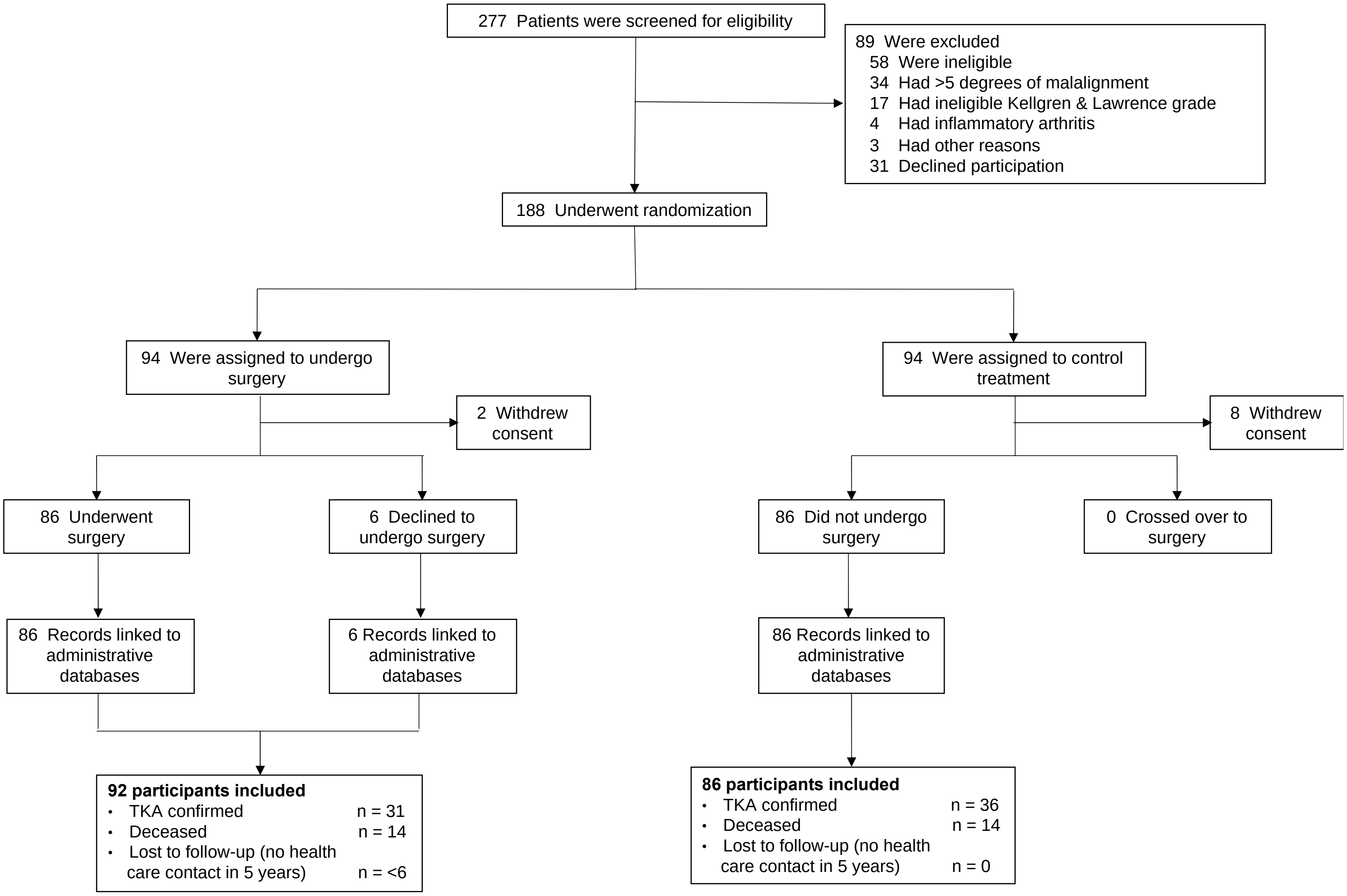

© 2024 Birmingham TB et al. *JAMA Network Open*.

Adapted from Kirkley A, Birmingham TB, Litchfield RB, Giffin JR, Willits KR, Wong CJ, Feagan BG, Donner A, Griffin SH, D'Ascanio LM, Pope JE, Fowler PJ. A randomized trial of arthroscopic surgery for osteoarthritis of the knee. N Engl J Med. 2008 Sep 11;359(11):1097-107. Copyright © 2008 Massachusetts Medical Society. Reprinted with permission. https://www.nejm.org/doi/full/10.1056/nejmoa0708333

© 2024 Birmingham TB et al. *JAMA Network Open*.

eTable 1. Description of Ontario Health Administrative Databases

All data used in this study were accessed at ICES (www.ices.on.ca), an independent, non-profit organization that uses routinely collected health administrative data to conduct health services and population health outcomes research. All residents of Ontario are included in Ontario's health, administrative data, and >99% of residents have a health card which is presented at each healthcare encounter. ICES links records within and across datasets using encrypted Ontario health card numbers. A more fulsome description of ICES, its governance structure and linkage practices are available elsewhere[1].

Name	Data Provider	Description
**Canadian Institute for Health Information Discharge Abstract Database (DAD)**	Canadian Institute for Health Information (CIHI)	The DAD is compiled by the Canadian Institute for Health Information (CIHI) and contains administrative, clinical (diagnoses and procedures/interventions), demographic, and administrative information for all admissions to acute care hospitals in Ontario. At ICES, consecutive DAD records are linked together to form 'episodes of care' among the hospitals to which patients have been transferred after their initial admission.<br><br>Prior to April 1, 2002, diagnoses (up to 16 on a given DAD record) are captured using the International Statistical Classification of Diseases, Injuries, and Causes of Death, 9th Revision (ICD-9) coding system and procedures (up to 10 on a given DAD record) are captured using the Canadian Classification of Diagnostic, Therapeutic, and Surgical Procedures (CCP) coding system. Following April 1, 2002, diagnoses (up to 25 on a given DAD record) are captured using the International Statistical Classification of Diseases and Related Health Problems, 10th Revision, Canada (ICD-10-CA) coding system and interventions (up to 20 on a given DAD record) are captured using the Canadian Classification of Health Interventions (CCI) coding system.<br><br>In a hospital medical record reabstraction study of 14,500 hospital discharges from 18 hospital sites between April 2002 and March 2004, DAD records were demonstrated to have excellent agreement (over 99%) for nonmedical information such as demographic and administrative data. Regarding diagnoses, median agreement between the original DAD records and the reabstracted records for the 50 most common most responsible diagnoses was 81% (Sensitivity 82%; Specificity 82%).(2) The corresponding median agreement for the 50 most frequently performed surgical procedures was 92% (sensitivity 95%, positive predictive value 91%).
**Canadian Institute for Health Information Same Day Surgery (SDS)**	Canadian Institute for Health Information (CIHI)	The SDS is compiled by the Canadian Institute for Health Information (CIHI) and contains administrative, clinical (diagnoses and procedures), demographic, and administrative information for all patient visits made to day surgery institutions in Ontario.<br><br>Prior to April 1, 2002, diagnoses (up to 16 on a given SDS record) were captured using the ICD-9 coding system and procedures (up to 10 on a given SDS record) were captured using the CCP coding system. Since April 1, 2002, diagnoses (up to 25 on a given SDS record) are captured using the ICD-10-CA coding system and interventions (up to 16 on a given SDS record) are captured using the CCI coding system.
**Ontario Health Insurance Plan (OHIP)**	Ministry of Health	The OHIP claims database contains information on inpatient and outpatient services provided to Ontario residents eligible for the province's publicly funded health insurance system by fee-forservice health care practitioners (primarily physicians) and "shadow billings" for those paid through non-fee-for-service payment plans.<br><br>Billing codes on the claims (OHIP fee codes) identify the care provider, their area of specialization and the type and location of service. OHIP billing claims also contain a 3-digit diagnosis code - the main reason for the service - captured using a modified version of the ICD, 8th revision coding system. OHIP claims are well completed, but the validity of the diagnosis coding is highly variable.(4)
**OHIP Registered Persons Database (RPDB)**	Ministry of Health	The OHIP RPDB provides basic demographic information (age, sex, location of residence, date of birth, and date of death for deceased individuals) for those issued an Ontario health insurance number. The RPDB also indicates the time periods for which an individual was eligible to receive publicly funded health insurance benefits and the best known postal code for each registrant on July 1st of each year.

Reference

1. Schull MJ, Azimaee M, Marra M, Cartagena RG, Vermeulen MJ, Ho M, & Guttmann A. ICES: data, discovery, better health. International *Journal of Population Data Science*. 2019; **4**(2).

© 2024 Birmingham TB et al. *JAMA Network Open*.

eTable 2. Variables

Variable	Data Source/Code	Definition/Description
**Age**	RPDB	Age at index date*
**Sex**	RPDB	Sex assigned at birth of the individual as recorded in the individual's health card. Reference category=Male
**Income Quintile**	RPDB	Neighbourhood income quintile
**Rurality**	RPDB	Living in towns or municipalities outside the outside the commuting zone of centres with population of 10,000 or more.
**Charlson Comorbidity Index**	DAD/ICD9, ICD 10	Within 2 years prior to index date*
**Knee Arthroscopy**	DAD/CCP Code 9232	EXCISION OF SEMILUNAR CARTILAGE OF KNEE
	DAD/CCP Code 9255	OTHER LOCAL EXCISION OR DESTRUCTION OF LESION OF JOINT, KNEE
	DAD/CCP Code 9285	ARTHROSCOPY, KNEE
	DAD/CCI Code 1VG52DA	Drainage, knee joint using endoscopic [arthroscopic] approach
	DAD/CCI Code 1VG80FY	Repair, knee joint endoscopic [arthroscopic] approach no tissue used (for repair) with meniscectomy [or meniscoplasty]
	DAD/CCI Code 1VG87DA	Excision partial, knee joint endoscopic [arthroscopic] approach, other joint excision [e.g. arthrectomy, chondrectomy, debridement] with or without synovectomy
	DAD/CCI Code 1VG87GB	Excision partial, knee joint endoscopic [arthroscopic] approach, synovectomy [dissection] alone
	DAD/CCI Code 1VK80DA	Repair, meniscus of knee endoscopic [arthroscopic] approach simple apposition technique (for tissue regeneration, e.g. suturing)
	DAD/CCI Code 1VK80GZ	Repair, meniscus of knee endoscopic [arthroscopic] approach with special incisional technique [e.g. multiple burr holes for revascularization] with no tissue used (for repair)
	DAD/CCI Code 1VK87DA	Excision partial, meniscus of knee using endoscopic [arthroscopic] approach
	DAD/CCI Code 1VK89DA	Excision total, meniscus of knee using endoscopic [arthroscopic] approach
	OHIP/Code E595	Arthroscopy - arthroscopy in association with surgery - same surgeon
	OHIP/Code R191	Synovial biopsy
	OHIP/Code R192	Trimming of plica, tissue, meniscus
	OHIP/Code R193	Removal of loose body, screw
	OHIP/Code R194	Resection of plica
	OHIP/Code R195	Lateral release
	OHIP/Code R204	Debridement - 1 compartment
	OHIP/Code R205	Debridement - more than 1 compartment
	OHIP/Code R207	Menisectomy
	OHIP/Code R208	Repair medial or lateral meniscus
	OHIP/Code R508	JOINT-RECONST-MENISCUS-SUTURE MEDIAL/LATERAL.
	OHIP/Code R687	Knee Arthroscopy set-up, includes when rendered synovial biopsy and/or resection or trimming of plica
	OHIP/Code Z218	Diagnostic arthroscopy (sole procedure)
**Hospitalization**	DAD	Discharges within 1 year prior to index date*
**Physician Consults**	OHIP	Consult billing within 1 year prior to index date*
**Total Knee Arthroplasty**	DAD/CCP Code 9341	JOINT-RECONST-KNEE HEMI-ARTHROPLASTY (SINGLE)
	DAD/CCI Code 1VG53LAPM	Implantation of internal device, knee joint uncemented single component prosthetic device
	DAD/CCI Code 1VG53LAPN	Implantation of internal device, knee joint uncemented using dual component prosthetic device
	DAD/CCI Code 1VG53LAPP	Implantation of internal device, knee joint uncemented tri component prosthetic device
	DAD/CCI Code 1VG53LASLN	Implantation of internal device, knee joint with synthetic material (e.g. bone paste, cement, Dynagraft, Osteoset) using cement spacer (temporary) [impregnated with antibiotics]
**Osteotomy**	OHIP/Code R289	BONES-RECONS-OSTEOTOMY-TIBIA & FIBULA-ADULT OR CHILD.
	OHIP/Code R971	MUSCULOSKEL.SYST.RECONST/DEFORM.SING.LEV.CORR.-CIRC.EXT.FIX
	OHIP/Code R972	MUSCULOSKEL.SYST.RECONST/DEFORM.DOUBLE.LEV.CORR.-CIR.EXT.FIX
**Meniscectomy**	DAD/ Code CCI 1VG80FY	Repair, knee joint endoscopic [arthroscopic] approach no tissue used (for repair) with meniscectomy [or meniscoplasty]
	DAD/ Code CCI 1VK80DA	Repair, meniscus of knee endoscopic [arthroscopic] approach simple apposition technique (for tissue regeneration, e.g. suturing)
	DAD/ Code CCI 1VK80GZ	

© 2024 Birmingham TB et al. *JAMA Network Open*.

	DAD/ Code CCI 1VK87DA	Repair, meniscus of knee endoscopic [arthroscopic] approach with special incisional technique [e.g. multiple burr holes for revascularization] with no tissue used (for repair)
	DAD/ Code CCI 1VK89DA	Excision partial, meniscus of knee using endoscopic [arthroscopic] approach<br>Excision total, meniscus of knee using endoscopic [arthroscopic] approach
	OHIP/ Code R192	Trimming of plica, tissue, meniscus
	OHIP/ Code R207	Menisectomy
	OHIP/ Code R208	Repair medial or lateral meniscus
	OHIP/ Code R508	JOINT-RECONST-MENISCUS-SUTURE MEDIAL/LATERAL.
**Body Mass Index**	Trial data to ICES (AUG 2019)	Body mass index at index date*
**Kellgren and Lawrence grade**	Trial data to ICES (AUG 2019)	Kellgren & Lawrence radiographic disease stage at index date*
**WOMAC score**	Trial data to ICES (AUG 2019)	Western Ontario & McMaster Universities Osteoarthritis Index at index date*
**Weight**	Trial data to ICES (AUG 2019)	Weight (in kilograms) at index date*
**Height**	Trial data to ICES (AUG 2019)	Height (in centimeters) at index date*
**Duration of symptoms**	Trial data to ICES (AUG 2019)	Duration of knee pain symptoms prior to index date*
**Lower limb anatomical alignment (degrees)**	Trial data to ICES (AUG 2019)	Lower limb anatomical alignment as assessed prior to index date*
**Symptoms of catching or locking**	Trial data to ICES (AUG 2019)	Symptoms of catching or locking in the knee on initial assessment (index date)*
**Joint effusion**	Trial data to ICES (AUG 2019)	Joint effusion in the knee on initial assessment (index date)*
**Pain with forced flexion**	Trial data to ICES (AUG 2019)	Pain with forced flexion of the knee joint on initial assessment (index date)*
**Tenderness at the tibiofemoral joint line**	Trial data to ICES (AUG 2019)	Medial joint line tenderness on palpation at the tibiofemoral joint on initial assessment (index date)*

***Index date =** First treatment date following randomization, from previous randomized trial: A Randomized Trial of Arthroscopic Surgery for Osteoarthritis of the Knee

© 2024 Birmingham TB et al. *JAMA Network Open*.

eTable 3. Full Baseline Demographic and Clinical Characteristics

No (%)	Arthroscopic Surgery (N=86)	Control (N=92)	Total (N=178)
Characteristics			
**Age**			
Mean ± SD	59.88 ± 9.85	58.10 ± 10.05	58.96 ± 9.97
Median (IQR)	61 (54-67)	57 (52-66)	59 (52-66)
**Female**	58 (67.4)	54 (58.7)	112 (62.9)
**Income quintile**			
Quintile 1	15 (17.4)	10 (10.9)	25 (14.0)
Quintile 2	13 (15.1)	12 (13.0)	25 (14.0)
Quintile 3	12 (14.0)	21 (22.8)	33 (18.5)
Quintile 4	19 (22.1)	24 (26.1)	43 (24.2)
Quintile 5	27 (31.4)	25 (27.2)	52 (29.2)
**Rural**	12 (14.0)	12 (13.0)	24 (13.5)
**Year of randomization**			
1999	18 (20.9)	17 (18.5)	35 (19.7)
2000	12 (14.0)	16 (17.4)	28 (15.7)
2001	16 (18.6)	10 (10.9)	26 (14.6)
2002	10 (11.6)	19 (20.7)	29 (16.3)
2003	10 (11.6)	10 (10.9)	20 (11.2)
2004	9 (10.5)	7 (7.6)	16 (9.0)
2005	11 (12.8)	13 (14.1)	24 (13.5)
Health Status			
**Weight** (kg)			
Mean ± SD	84.75 ± 17.97	91.19 ± 17.14	88.10 ± 17.79
Median (IQR)	82 (73-94)	91 (79-104)	84 (75-100)
**Height** (cm)			
Mean ± SD	167.73 ± 10.22	170.35 ± 9.59	169.09 ± 9.96
Median (IQR)	166 (160-173)	168 (163-177)	168 (163-175)
**Body Mass Index**			
Mean ± SD	30.24 ± 6.31	31.60 ± 6.70	30.95 ± 6.53
Median (IQR)	29 (25-34)	31 (27-35)	30 (26-35)
**Duration of symptoms** (months)			
Mean ± SD	41.53 ± 73.34	47.08 ± 69.36	44.45 ± 71.12
Median (IQR)	12 (8-36)	21 (9-54)	18 (9-48)
**Kellgren and Lawrence grade**			
2	36 (41.9)	<=45	<=80
3	45 (52.3)	45 (48.9)	90 (50.6)
4	<=5 [a]	<=5 [a]	9 (5.1)
Missing	<=5 [a]	0 (0.0)	<=5 [a]
**Lower limb anatomical alignment** (degrees)			
Mean ± SD	1.22 ± 3.94	1.15 ± 3.41	1.18 ± 3.66
Median (IQR)	1 (-2-4)	1 (-1-4)	1 (-2-4)
**Symptoms of catching or locking**	38 (44.2)	49 (53.3)	87 (48.9)
**Joint effusion**	72 (83.7)	76 (82.6)	148 (83.1)
**Pain with forced flexion**	56 (65.1)	62 (67.4)	118 (66.3)
**Tenderness at the tibiofemoral joint line**	75 (87.2)	81 (88.0)	156 (87.6)
**Baseline WOMAC total score** [b]			
Mean ± SD	1,066.46 ± 551.39	1,169.95 ± 482.58	1,120.56 ± 517.67
Median (IQR)	1,102 (665-1,546)	1,184 (811-1,497)	1,154 (759-1,503)
Comorbidities			
**Charlson Comorbidity Score**			
Mean ± SD	0.56 ± 0.88	0.33 ± 0.65	0.43 ± 0.75
Median (IQR)	0 (0-1)	0 (0-1)	0 (0-1)
Reported score available	9 (10.5)	12 (13.0)	21 (11.8)
No Hospitalizations	77 (89.5)	80 (87.0)	157 (88.2)
Healthcare system utilization in the past year			
**Hospitalizations**			
Mean ± SD	0.02 ± 0.15	0.07 ± 0.29	0.04 ± 0.23
**Physician consults**			
Mean ± SD	15.36 ± 8.74	18.48 ± 12.08	16.97 ± 10.69
Median (IQR)	14 (10-20)	14 (10-24)	14 (10-22)
0			
1-10	26 (30.2)	27 (29.3)	53 (29.8)

© 2024 Birmingham TB et al. *JAMA Network Open*.

11+	60 (69.8)	65 (70.7)	125 (70.2)

Abbreviations: IQR = inter-quartile range, N = sample size, SD = standard deviation

All patients had a clinical diagnosis of knee osteoarthritis according to the American College of Rheumatology Criteria as described by Altman (1986). Clinical knee osteoarthritis is defined as knee pain and 3 of the 6 following criteria: morning stiffness < 30 minutes, age > 50 years, crepitus, bony tenderness, bony enlargement and/or no palpable warmth.

[a] In accordance with ICES privacy policies, cell sizes less than or equal to five cannot be reported.

[b] WOMAC = Western Ontario and McMaster Universities Osteoarthritis Index (WOMAC) includes three subscales (pain, stiffness, and physical function) calculated from 24 questions. Values range from 0 (no disease) to 2400 (more severe disease).

Adapted from Kirkley A, Birmingham TB, Litchfield RB, Giffin JR, Willits KR, Wong CJ, Feagan BG, Donner A, Griffin SH, D'Ascanio LM, Pope JE, Fowler PJ. A randomized trial of arthroscopic surgery for osteoarthritis of the knee. N Engl J Med. 2008 Sep 11;359(11):1097-107. Copyright © 2008 Massachusetts Medical Society. Reprinted with permission. https://www.nejm.org/doi/full/10.1056/nejmoa0708333

eTable 4. Use of Medical, Physical, and Surgical Therapy in the Patients

No (%)	Arthroscopic Surgery (N=92)	Control (N=86)
**Therapy**		
Medical therapy		
Nonsteroidal anti-inflammatory drugs	53 (58)	48 (56)
Acetaminophen	53 (58)	43 (50)
Chondroitin sulfate or glucosamine	28 (30)	25 (29)
Hyaluronic acid injection	39 (42)	33 (38)
Physical therapy		
Patients participating	88 (96)	77 (90)
No. of visits by participating patients	9.3 ± 5.1	8.0 ± 5.7
Use of brace	<=5 [a]	<=5 [a]
Surgical therapy [b]		
Débridement of articular cartilage	83 (97)	N/A
Débridement or partial resection of meniscus	70 (81)	N/A
Repair of meniscus	0 (0)	N/A
Excision of osteophytes	8 (9)	N/A
Removal of loose bodies	12 (14)	N/A

[a] In accordance with ICES privacy policies, cell sizes less than or equal to five cannot be reported.
[b] The percentages are based on 86 patients rather than 92 because 6 patients who were assigned to surgery declined the procedure

Adapted from Kirkley A, Birmingham TB, Litchfield RB, Giffin JR, Willits KR, Wong CJ, Feagan BG, Donner A, Griffin SH, D'Ascanio LM, Pope JE, Fowler PJ. A randomized trial of arthroscopic surgery for osteoarthritis of the knee. N Engl J Med. 2008 Sep 11;359(11):1097-107.
Copyright © 2008 Massachusetts Medical Society. Reprinted with permission. https://www.nejm.org/doi/full/10.1056/nejmoa0708333

© 2024 Birmingham TB et al. *JAMA Network Open*.

eTable 5. Cox Proportional Hazards Regression Model for Primary and Secondary Outcomes, With Knee Arthroscopy During Long-Term Follow-Up (Crossovers) Used as a Time-Varying Covariate

Variable	Reference	Hazard Ratio (95% Confidence Interval)	
		**Total knee arthroplasty on the study knee (N=178)**	**Total knee arthroplasty or high tibial osteotomy on either knee (N=178)**
**Arthroscopic Surgery**	Control	0.88 (0.53-1.44)	1.08 (0.69-1.68)
**Age** (per 10 years)	-	1.16 (0.90-1.51)	1.12 (0.88-1.41)
**Sex**	Male	1.41 (0.80-2.46)	1.38 (0.85-2.25)
**Body mass index** (per 5 kg/m^2)		1.07 (0.89-1.29)	1.03 (0.87-1.22)
**Kellgren and Lawrence grade > 2**	Kellgren and Lawrence grade 2	2.04 (1.19-3.48)	1.90 (1.21-3.01)
**Baseline WOMAC** [a] (per 200 points)		1.01 (0.91-1.12)	1.01 (0.92-1.11)

We conducted a complete case analysis where <6 participants were excluded in analysis due to missing covariates (ie, body mass index, Kellgren and Lawrence grade and/or baseline WOMAC).

For total knee arthroplasty on the study knee as the outcome, 13 of 86 No Knee Arthroscopy patients had a scope during long-term follow-up. For total knee arthroplasty or high tibial osteotomy on either knee as the outcome, 12 of 86 No No Knee Arthroscopy patients had a scope in follow-up.

<6 participants who switched from No Knee Arthroscopy to Knee Athroscopy (exposure) had missing laterality which were coded as same knee as study.

[a] WOMAC = Western Ontario and McMaster Universities Osteoarthritis Index (WOMAC) includes three subscales (pain, stiffness, and physical function) calculated from 24 questions. Values range from 0 (no disease) to 2400 (more severe disease).

© 2024 Birmingham TB et al. *JAMA Network Open*.

eTable 6. Cox Proportional Hazards Regression Model for Primary and Secondary Outcomes, With Knee Arthroscopy During Long-Term Follow-Up for the As-Treated Analysis

**Variable**	**Reference**	**Hazard Ratio (95% Confidence Interval)**	
		**Total knee arthroplasty on the study knee (N=178)**	**Total knee arthroplasty or high tibial osteotomy on either knee (N=178)**
**Arthroscopic Surgery**	Control	0.98 (0.60-1.62)	0.94 (0.60-1.46)
**Age** (per 10 years)	-	1.17 (0.90-1.51)	1.10 (0.87-0.41)
**Sex**	Male	1.43 (0.82-2.50)	1.35 (0.82-2.20)
**Body mass index** (per 5 kg/m^2)		1.07 (0.89-1.29)	1.03 (0.87-1.22)
**Kellgren and Lawrence grade > 2**	Kellgren and Lawrence grade 2	2.05 (1.20-3.51)	1.91 (1.20-3.02)
**Baseline WOMAC** [a] (per 200 points)		1.01 (0.91-1.12)	1.02 (0.93-0.75)

We conducted a complete case analysis where <6 participants were excluded in analysis due to missing covariates (ie, body mass index, Kellgren and Lawrence grade and/or baseline WOMAC).

[a] WOMAC = Western Ontario and McMaster Universities Osteoarthritis Index (WOMAC) includes three subscales (pain, stiffness, and physical function) calculated from 24 questions. Values range from 0 (no disease) to 2400 (more severe disease).

© 2024 Birmingham TB et al. *JAMA Network Open*.

eTable 7. Cox Proportional Hazards Regression Model for Primary and Secondary Outcomes in Patients With a Kellgren and Lawrence Grade of 2 (n = 78)

**Variable**	**Reference**	**Hazard Ratio (95% Confidence Interval)**	
		**Total knee arthroplasty on the study knee (N=78)**	**Total knee arthroplasty or high tibial osteotomy on either knee (N=78)**
**Arthroscopic Surgery**	Control	0.79 (0.33-1.92)	0.84 (0.40-1.76)
**Age** (per 10 years)	-	1.10 (0.65-1.84)	1.11 (0.72-1.71)

© 2024 Birmingham TB et al. *JAMA Network Open*.

eTable 8. Cox Proportional Hazards Regression Model for Primary and Secondary Outcomes in Patients Experiencing Knee Catching or Locking (n = 87)

**Variable**	**Reference**	**Hazard Ratio (95% Confidence Interval)**	
		**Total knee arthroplasty on the study knee (N=87)**	**Total knee arthroplasty or high tibial osteotomy on either knee (N=87)**
**Arthroscopic Surgery**	Control	1.13 (0.60-2.13)	1.23 (0.69-2.18)
**Age** (per 10 years)	-	1.21 (0.86-1.70)	1.15 (0.85-1.57)
**Sex**	Male	1.46 (0.69-3.11)	1.49 (0.75-2.96)
**Kellgren and Lawrence grade > 2**	Kellgren and Lawrence grade 2	1.62 (0.82-3.20)	1.14 (0.63-2.07)

© 2024 Birmingham TB et al. *JAMA Network Open*.
