# Supplementary material for: Incidence of Total Knee Arthroplasty After Arthroscopic Surgery for Knee Osteoarthritis: A Secondary Analysis of a Randomized Clinical Trial
Source: JAMA Netw Open. 2024 Apr 18;7(4):e246578. doi: 10.1001/jamanetworkopen.2024.6578 (PMC12503430; doi:10.1001/jamanetworkopen.2024.6578)
Supplement: Supplement 3. — Data Sharing Statement [file jamanetwopen-e246578-s003.pdf]

Data Sharing Statement

Birmingham. Incidence of Total Knee Arthroplasty After Arthroscopic Surgery for Knee Osteoarthritis. *JAMA Netw Open*. Published April 18, 2024.
doi:10.1001/jamanetworkopen.2024.6578

Data
Data available: No

Additional Information
Explanation for why data not available: The datasets used in this study are held securely in coded form at ICES. While legal data sharing agreements between ICES and data providers (e.g., health care organizations and government) prohibit ICES from making the data set publicly available, access may be granted to those who meet pre-specified criteria for confidential access, available at https://www.ices.on.ca/DAS (email: das@ices.on.ca). The full data set creation plan and underlying analytic code are available from the authors upon request, understanding that the computer programs may rely upon coding templates or macros that are unique to ICES and are therefore either inaccessible or may require modification.
